# Switching Cathodic/Anodic Electrochemiluminescence of Ru(bpy)_3_^2+^ Precisely via Homogeneous Nickel Nanoparticles Crystal Facets Sites Modulated ORR/OER

**DOI:** 10.1002/EXP.20250036

**Published:** 2025-08-30

**Authors:** Zixin Deng, Zhizhi Xiang, Shu Zhu, Yuchan Zhang, Yu Du, Shijun Wang, Ziqi Kang, Zixu Wang, Xuehao Tong, Yangkun Liu, Lingfang Jiang, Anna Malashicheva, Hao Sun, Feng Dong, Guixue Wang, Chenzhong Li, Guangchao Zang

**Affiliations:** ^1^ Academic Affairs Office The Second Affiliated Hospital of Chongqing Medical University Chongqing China; ^2^ JinFeng Laboratory Chongqing China; ^3^ Lab Teaching & Management Center Chongqing Medical University Chongqing China; ^4^ College of Biomedical Engineering Chongqing Medical University Chongqing China; ^5^ Institute of Cytology Russian Academy of Science Petersburg Russia; ^6^ UCL Division of Medicine University College London London United Kingdom; ^7^ Department of Clinical Laboratory The Second Affiliated Hospital, Army Medical University Chongqing China; ^8^ Key Laboratory for Biorheological Science and Technology of Ministry of Education, National Local Joint Engineering Lab for Vascular Implants, College of Bioengineering Chongqing University Chongqing China; ^9^ School of Biosciences and Technology Chengdu Medical College Chengdu China; ^10^ Bioelectronics and Biosensors Center, School of Medicine Chinese University of Hong Kong Shenzhen China

**Keywords:** crystal facets, electrochemiluminescence, ROS intermediate, Ru(bpy)_3_^2+^, sensing

## Abstract

Reactive oxygen species (ROS) have gained increasing attention in electrochemiluminescence (ECL) as endogenous co‐reactants, yet their application in the most widely used tris(bipyridine)‐ruthenium(II) system remains limited due to the scarcity of suitable co‐reactant accelerators (CRAs) with selective oxygen reduction reaction (ORR) and oxygen evolution reaction (OER) catalytic activity. Here, this work reports a series of facet‐tunable homogeneous NiNPs catalysts, which can stimulate ECL at distinguishable cathodic/anodic potentials in tris(bipyridine)‐ruthenium(II) system. Experimental studies and theoretical calculation results reveal that the Ni(1 1 0) surface, with its lower charge density, impedes the fourth step of 4e^−^ ORR, thus favoring 2e^−^ pathway and consequently promoting substantial ROS generation and ECL at the cathode. Conversely, the Ni(1 1 1) and (2 0 0) surface prompt robust and stable anodic ECL via hydroxyl radical by controlling the OER. These excellent CRAs link cathodic/anodic ECL with ORR/OER, offering a novel strategy for precisely designing predictable non‐precious metal CRAs. Furthermore, sensitive immunosensors were developed using these CRAs, demonstrating successful application in potential‐resolved ECL analysis for practical purposes.

## Graphical abstract

Mechanistic Illustration of the facet modulated Ru(bpy)3^2+^ cathodic/anodic ECL system. O_2_ molecules undergo ORR at the cathode, while H_2_O molecules undergo OER at the anode on different catalyst facets. Ni/NG‐1 exhibits high OER but low ORR activity, generating radicals that react with Ru(bpy)_3_^2+^ to enhance cathodic ECL. In contrast, Ni/NG‐2 shows high ORR but low OER activity, thus promoting anodic ECL through a similar radical‐assisted pathway.

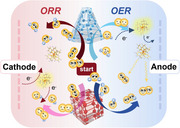

## 1 Introduction

Electrochemiluminescence (ECL) is a photoexcitation phenomenon based on the electron transfer between free radicals generated during an electrochemical reaction and reactive luminophores [1, 2]. In the expanding field of ECL, tris(bipyridine)‐ruthenium(II) (Ru(bpy)_3_^2+^) has become one of the most widely studied luminophores, due to its exceptional stability, high quantum yield, and ability to achieve both temporal and spatial resolution, making it valuable in both basic research and commercial applications, especially in vitro diagnosis [3, 4, 5]. Traditional Ru(bpy)_3_^2+^ ECL relies on exogenous co‐reactants, such as tripropylamine (TPrA) [6] and hydrogen peroxide (H_2_O_2_) [7], which introduce significant limitations. Traditional TPrA requires extremely high concentrations (about 50 mM) to achieve effective luminescence, but high concentrations of TPrA may interfere with the reaction system and damage samples [8], leading to electrode degradation and unstable signal output, while the inherent instability of H_2_O_2_ under ambient conditions restricts the practicality of the system [6, 7]. Recently, reactive oxygen species (ROS) have emerged as a promising endogenous co‐reactant for ECL due to their dynamic stability. ROS are primarily generated via two electrochemical pathways: oxygen reduction reaction (ORR) and oxygen evolution reaction (OER). These reactions inherently offer potential‐resolving capabilities, as their distinct redox potentials allow selective ROS generation under controlled voltages. Moreover, this method possesses significant advantages, particularly the ability to maintain the dynamic stability of the Ru(bpy)_3_^2+^ system by modulating the reaction rate, thereby enabling potential resolution [9, 10, 11, 12]. Nevertheless, the low spontaneous conductivity of ORR/OER and insufficient ROS accumulation limit its practical application. While highly selective catalysts in the Ru(bpy)_3_^2+^‐ROS system hold promise for enhancing the ECL mechanism by modulating ORR and OER processes, the scarcity of such catalysts limits its application [13, 14, 15, 16, 17].

Recently, nickel nanoparticles (NiNPs) composited with graphene oxide (GO) have been identified as promising bifunctional catalysts for both ORR and OER, demonstrating high efficiency under specific conditions, which are particularly attractive due to their enhanced electrochemical performance, stability, and tunable surface properties [18, 19, 20, 21]. In this study, we synthesized a series of NiNPs with fine‐tuned distinct crystal facets, supported on nitrogen‐doped graphene oxide (N‐doped GO). From this series, we selected two specific variants—denoted Ni/NG‐1 and Ni/NG‐2—as model catalysts, to precisely modulate the formation of specific radicals in both ORR and OER processes, ultimately enhancing potential‐resolved Ru(bpy)_3_^2+^ ECL [22, 23, 24, 25]. Ni/NG‐1, with its Ni(1 1 0) crystal surface, exhibits a lower surface charge density, which restricts electron transfer, favoring the 2^−^electron pathway in the ORR. Density functional theory (DFT) calculations and in situ infrared (in situ IR) spectroscopy show that the fourth step of the ORR is the rate‐determining step for Ni/NG‐1, resulting in the formation of massive superoxide radicals (•O_2_H) and hydroxyl radicals (•OH), which significantly enhance cathodic ECL. In contrast, Ni/NG‐2 promotes anodic ECL by catalyzing the OER process, which generates •OH at the anode. The potential‐resolved ECL enhancement achieved through dual cathodic and anodic co‐reactant accelerators (CRAs) demonstrates an effective strategy for ultrasensitive, multi‐signal biodetection. Using carcinoembryonic antigen (CEA) and carbohydrate antigen 125 (CA125) as model targets, we showcased the ability of this approach to improve detection sensitivity and reliability.

This study provides valuable insights into the relationship between ORR/OER processes and cathodic/anodic ECL excitation in the Ru(bpy)_3_^2+^‐ROS system. Specifically, a series of precise NiNPs catalysts with tailored crystal facets was developed, which breaks through the bottleneck of potential‐resolved co‐reactants, enabling efficient and controlled cathodic and anodic ECL. Additionally, we delve deeper into the reaction mechanisms within the ROS‐Ru(bpy)_3_^2+^ system, examining electron pathways, light emission processes, and the types of intermediate ROS species formed at different potentials. Our work also elucidates the surface effect mechanisms and dynamics of NiNPs in regulating ROS, with insights derived from DFT calculations. These guide the rapid screening of optimal catalysts and help us to propose design principles for non‐precious metal NP catalysts optimized for ORR/OER reactions. Through the above efforts, a novel dual‐signal strategy for an enhanced ECL‐based analysis system was developed, demonstrating high performance in ultrasensitive immunoassays and offering a robust platform that suggests significant potential for advancing ECL analysis and furthering exploration in catalyst design and ECL mechanism studies.

## 2 Synthesis and Characterization of the Ni/NG Catalysts

**FIGURE 1:**
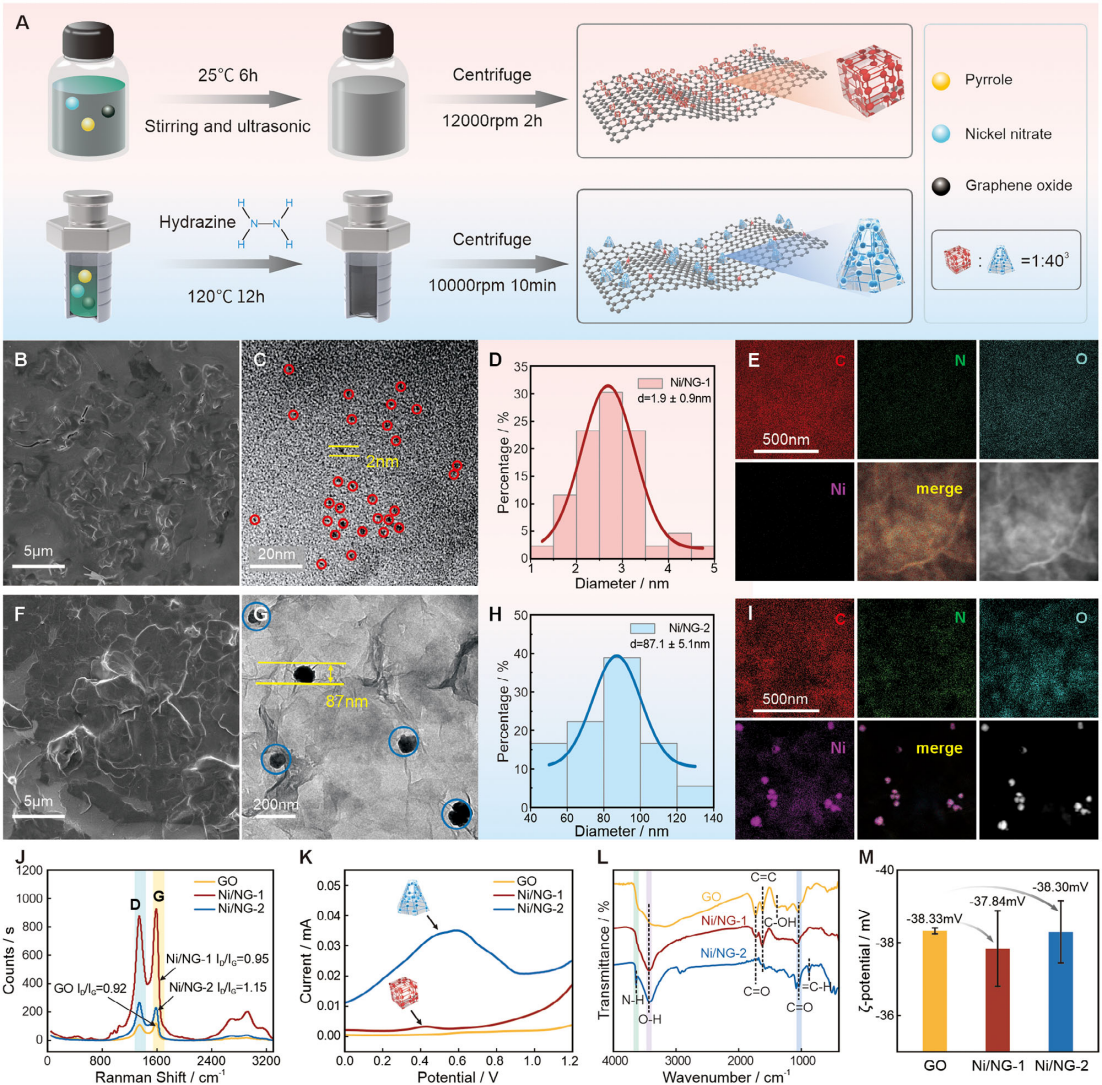
Synthesis and characterization of Ni/NG. (A) Schematic illustration of the synthesis of Ni/NGs. (B) SEM, (C) TEM, (D) size distribution of NiNPs, and (E) EDS mapping of Ni/NG‐1 (B–E) and Ni/NG‐2 (F–I). (J) Raman spectrum. (K) DPV test. (L) FTIR spectroscopy. (M) Zeta potential.

Figure 1A shows the preparation procedures of the two Ni/NGs. GO absorbs hydrated Ni(NO_3_)_2_ through host–guest interactions via a gentle stirring‐reduction to form Ni/NG‐1. Ni/NG‐2 with pyrrole and hydrazine hydrate was synthesized by a simple one‐pot‐method. A large‐scale scanning electron microscopy (SEM) shows that GO has flexible two‐dimensional sheet structures without noticeable phase separation (Figure 1B,F). Transmission electron microscopy (TEM) (Figure 1C,G; Figures S1 and S2) further reveals the uniform distribution of NiNPs on the surface of the GO sheets. The particle size distribution of the two NiNPs obtained by TEM is shown in Figure 1D,H. Energy‐dispersive spectroscopy (EDS) mappings (Figure 1E,I; Figures S3 and S4; Table S1) demonstrate the homogeneous dispersion of C, N, O, and Ni across the surfaces. Raman spectra and electrochemical differential pulse voltammetry (DPV) (Figure 1J,K) reveal that the incorporation of NiNPs increases defect density within the GO layers, while also confirming the successful synthesis of NiNPs on the GO surface [26]. Fourier transform infrared (FTIR) spectroscopy (Figure 1L) discloses Ni/NG‐1 has an excess ─OH stretching absorption peak at 1718 cm^−1^ compared to GO, whereas Ni/NG‐2 possesses abundant ─COOH, ─NH_3_, and ─OH. Meanwhile, high‐resolution X‐ray photoelectron spectroscopy (XPS) (Figures S5–S7) shows that N acts as a linking agent that connects the metal NPs. In addition, although the introduction of metal nanoparticles neutralizes the charges on GO and affects its dispersibility, the electrostatic repulsion of the metal nanoparticles significantly increases the layered structure of GO. As a result, the final decrease in Zeta potential is very slight; the absolute value of the zeta potential indicates that all three dispersion systems are stable (Figure 1M). The above results demonstrate that the synthesized materials exhibit a uniform structure, excellent dispersibility, and good stability, which are advantageous for both the mechanistic study and practical application of ECL.

## 3 Evaluation of Ni/NG's ECL and ORR/OER Performance

**FIGURE 2:**
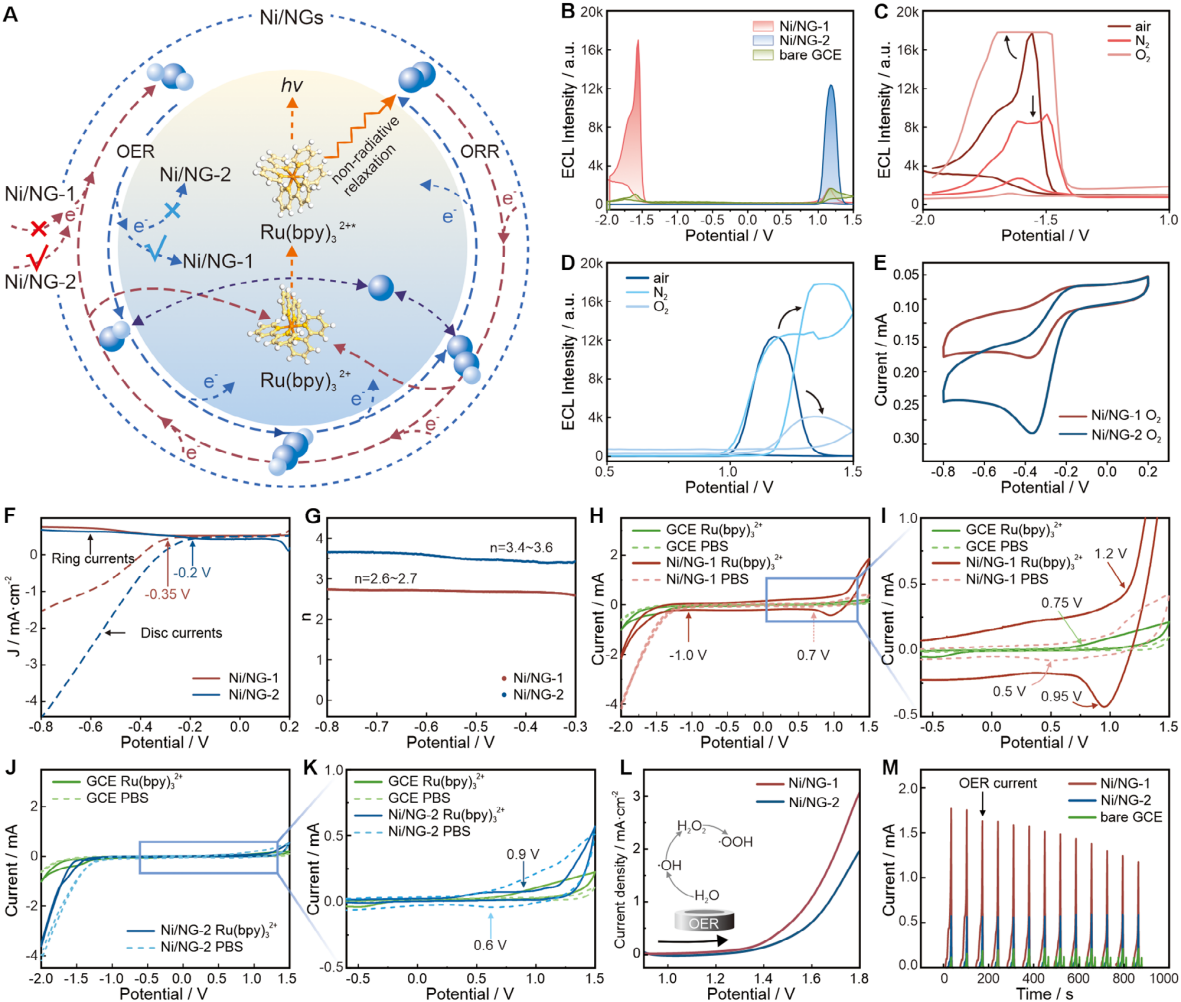
ECL and ORR/OER performance of Ni/NG. (A) ORR and OER reaction pathways and coupled Ru(bpy)_3_^2+^ ECL system. (B) ECL curves of Ni/NG in a 1 mM Ru(bpy)_3_^2+^ solution. ECL tests of Ni/NG‐1 (C) and Ni/NG‐2 (D) in a 1 mM Ru(bpy)_3_^2+^ solution (pH 6) under the varied atmospheres. (E) CV curves of Ni/NG in 0.1 M KOH in O_2_. (F) Ring electrode polarization curve (solid line) and disc electrode polarization curve (dashed line) at 1600 rpm and 5 mV s^−1^ rate. (G) Electron transfer number. (H) CV curves of Ni/NG‐1 in PBS and Ru(bpy)_3_^2+^ solutions. (I) The enlarged picture of CV curves in (H) from −0.5 to 1.5 V. (J) CV curves of Ni/NG‐2 in PBS and Ru(bpy)_3_^2+^ solutions. (K) The enlarged picture of CV curves in (J) from −0.5 to 1.5 V. (L) LSV scan and (M) current−time curves of Ni/NG in 1 mM Ru(bpy)_3_^2+^.

ORR and OER are two crucial processes that generate ROS in a stepwise manner (Figure 2A). Catalysts influence the intermediate steps of these processes, affecting ROS generation and subsequently promoting Ru(bpy)_3_^2^⁺ ECL emission at specific potentials (Figure 2B) [27]. To demonstrate the essential role of O_2_ as a co‐reactant precursor, the ECL performance was evaluated under varied atmospheres (Figure 2C,D). In air‐saturated Ru(bpy)_3_^2+^, the Ni/NG catalysts exhibit excellent ECL at −1.7 and 1.2 V, respectively. And in the oxygen environment, the ECL luminescence value of Ni/NG‐1 increased compared to the “air group,” even exceeding the instrumental measurement limit, showing a “flat peak” (Figure 2C), while the luminescence value of Ni/NG‐2 decreased significantly compared with that of the “air group” (Figure 2D). This suggests that O_2_ further promotes the cathodic luminescence of Ni/NG‐1/Ru(bpy)_3_^2+^ system and inhibits the anodic luminescence of Ni/NG‐2/ Ru(bpy)_3_^2+^ system. The ORR activity of the Ni/NG catalysts was evaluated via cyclic voltammetry (CV) scans (Figure 2E). Compared to Ni/NG‐1, Ni/NG‐2 shows a distinct O_2_ reduction peak at −0.35 V with a larger enclosed area, suggesting a stronger ORR catalytic activity (Figure S8). For Ni/NG‐1, the associated ECL signal attenuates under N_2_‐saturated Ru(bpy)_3_^2+^, where the absence of O_2_ hinders the electrocatalytic reaction. In contrast, the anodic ECL of Ni/NG‐2 reduces in O_2_, indicating that O_2_ is noxious to the anodic ECL (Figure S9).

ORR polarization curves were recorded at a scan rate of 5 mV s^−1^ (Figure 2F). The onset potential of Ni/NG‐1 is −0.35 V versus Ag/AgCl, which is close to the thermodynamic limit of the 2e^−^ ORR, and lower than that of Ni/NG‐2 (−0.2 V). According to the Koutecky−Levich (K‐L) plots, the electron‐transfer numbers (*n*) for Ni/NG‐1 and Ni/NG‐2 are ≈2.6–2.7 and ≈3.5–3.6, with the H_2_O_2_ yields of 25% and 65%, respectively (Figure 2G; Figure S10) [28]. This indicates that Ni/NG‐1 catalyzes ORR mainly through a 2e^−^ transfer pathway with superior ORR activity to that of Ni/NG‐1. Additionally, the CV current changes in Ru(bpy)_3_^2+^ demonstrate that Ni/NG‐1 and Ni/NG‐2 exhibit minimal reduction peaks at 0.5 and 0.6 V in PBS, compared to the bare glassy carbon electrode (GCE) (Figure 2H–K), which suggests that ORR occurs in both environments. However, when Ni/NG‐1 and Ru(bpy)_3_^2+^ coexist, a pronounced ORR reduction peak emerges at 0.95 V, confirming that their coexistence accelerates the ORR and promotes ROS generation (Figure 2I). When Ni/NG‐2 and Ru(bpy)_3_^2+^ are present, the reduction peaks of the CV curve become indistinct (Figure 2K), suggesting that other reactions occur, leading to more complex intermediate species that obscure the original peak [29].

These CV curves also show the OER process of the Ni/NG catalysts. Comparing the GCE in 1 mM Ru(bpy)_3_^2+^ and PBS at the anode, the background current increases at 0.75 V due to the oxidation of Ru(bpy)_3_^2+^, and then from the OER. Ni/NG‐1 exhibits an OER potential of 0.7 V in PBS, and positive current starts at −1.0 V in Ru(bpy)_3_^2+^. The oxidation current of Ru(bpy)_3_^2+^ with water begins to overlap at 1.2 V (Figure 2H,I), causing the disappearance of the OER oxidation shoulder peak. In contrast, Ni/NG‐2 has an OER onset potential of approximately 0.9 V in both PBS and Ru(bpy)_3_^2+^ solutions (Figure 2J,K), reflecting its relatively weaker OER capability. Furthermore, CV (Figure S11) and linear sweep voltammetry (LSV) scans (Figure 2L) from forward potential scanning demonstrate that Ni/NG‐1 significantly outperforms Ni/NG‐2 in terms of OER catalytic performance, as evidenced by the peak at 1.5 V corresponding to typical OER current (Figure 2M) [30, 31]. Moreover, the surface‐sensitive nature of the ECL reaction leads to the suppression of anodic ECL because of a non‐radiative relaxation pathway when Ru(bpy)_3_^2+^ reacts with oxygen in aqueous solution. Ni/NG‐2 leverages its weaker OER catalytic performance to mitigate the energy losses caused by this pathway, thereby enhancing the anodic ECL (Figure 2A).

## 4 Identification of Active Facets

**FIGURE 3:**
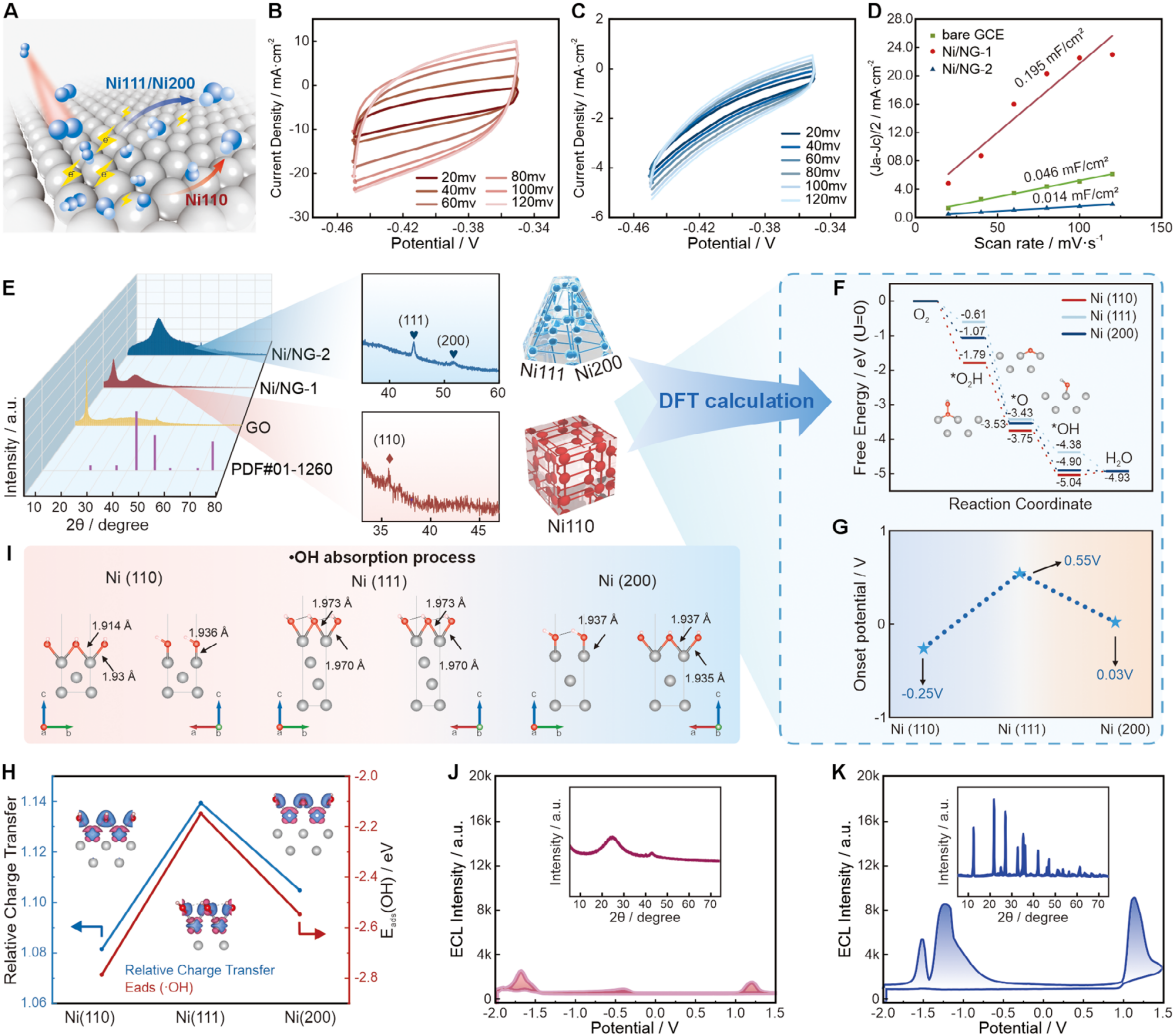
DFT‐assisted calculation verification. (A) Schematic diagram of the theoretical reaction pathways on different crystal facets. Electrochemical active surface area (ECSA) test of (B) Ni/NG‐1 and (C) Ni/NG‐2. (D) Electrochemical surface areas. (E) XRD of GO and Ni/NG, and the corresponding structural projection diagram of the two NiNPs. (F) Free energy profile for the ORR mechanism for Ni/NG at 0 V. (G) The theoretical onset potentials for different crystal surfaces catalyzing 4e^−^ ORR through DFT kinetic simulations. (H) Relative charge transfer and •OH adsorption energy of Ni (1 1 0), Ni (1 1 1), and Ni (2 0 0). (I) Differential charge density and the mutual distance between radical molecules and Ni during the absorption process of Ni (1 1 0), Ni (1 1 1), and Ni (2 0 0). (J) X‐ray diffraction (XRD) and ECL performance of Ni/NG‐1′. (K) XRD and ECL performance of Ni/NG‐2′.

To confirm NiNPs’ role as active moieties in promoting Ru(bpy)_3_^2+^ ECL, the ECL signals of bare GCE, GO and N‐doped graphene (Ni/NG substrate) were tracked (see method in supporting information). The Ni/NG substrate exhibited interfering cathodic or anodic ECL signals (Figure S12). Meanwhile, to eliminate the influence of surface area on catalytic activity, the catalyst size and electrochemically active surface area (ECSA) were evaluated (Figure 3B–D), which are key parameters in modern electrocatalyst design. Ni/NG‐1, with smaller NPs, exhibited a larger specific surface area and a double‐layer capacitance (Cdl) value of 0.195 mF cm^−^^2^, far exceeding that of Ni/NG‐2 (Cdl = 0.014 mF cm^−^^2^) (Figure 3D). These results align with previous size distribution outcomes (Figures S1C and S2C). However, the ultimate ORR mass activity results from the interplay between the ECSA and the specific activity of the ORR [32, 33, 34].

We then recorded X‐ray diffraction (XRD) to explore the other factors that essentially affect those parameters. Based on the comparison with XRD of the Ni PDF #01‐1260 cards [35, 36], the distinct peak at 35.56° for Ni/NG‐1 corresponds to the Ni (1 1 0) facet, and the peaks at 43.66° and 51.17° for Ni/NG‐2 correspond to the Ni (1 1 1) and Ni (2 0 0) facets, respectively. Structural predictions are graphed based on XRD results (Figure 3E). Next, we applied DFT methods to investigate the kinetics of the ORR on the active sites of the NiNPs [37]. We built surface models based on the XRD results (Figure S13), and assessed the adsorption capabilities of the Ni (1 1 0), Ni (1 1 1), and Ni (2 0 0) surfaces for •O_2_H, •O, and •OH species [38], along with system stability. The results in Figure 3F reveal that the most positive Δ*G* (change in free energy) in the simulated ORR kinetics occurs during the fourth step (*OH + H^+^ + e^−^ →H_2_O*), which serves as the rate‐determining step of ORR under the catalysis of all three dominant NiNP surfaces [39]. Notably, the Δ*G* for the Ni (1 1 0) facet in the fourth step is the highest (0.25 eV), indicating that it has the most negative theoretical onset potential (−0.25 V) (Figure 3G) and suggesting a highly constrained catalytic process [40]. Furthermore, we calculated the adsorption energy of the •OH radical (Figure 3H) and found that Ni (1 1 0) exhibits the lowest charge transfer to •OH(ads), suggesting that the electron transfer from Ni (1 1 0) to •OH is the weakest [41]. These findings suggest that the atomic arrangement on different crystal facets alters surface charge distribution, leading to variations in relative charge transfer [42].

Additionally, the adsorption energy of •OH on the Ni (1 1 0) surface is the highest, with the closest interaction distances between radical molecules and Ni atoms (1.914 and 1.936 Å) (Figure 3I) [21, 43]. In the fourth step, H^+^ migrates to the catalytic surface and binds to the •OH(ads) to form H_2_O^*^(ads). Due to the strong adsorption of •OH on the catalyst, this desorption step becomes difficult. Therefore, •OH can be selectively generated and accumulate on the Ni (1 1 0) surface, enhancing the cathodic ECL with Ru(bpy)_3_^+^. In contrast, Ni (1 1 1) and Ni (2 0 0) have a smaller adsorption energy of •OH, which is favorable for facilitating the following process of H_2_O^*^ dissociation to generate H_2_O. This can be attributed to compact atomic arrangement on Ni(1 1 1) and Ni(2 0 0), leading to a greater surface charge density of Ni/NG‐2, which facilitates electron transfer between the surface Ni atoms and the adsorbed molecules. A “substitution‐style” approach was employed to evaluate the effect of synthetic methods on crystal surfaces and catalytic performance. Specifically, materials from Ni/NG‐1 were utilized via the Ni/NG‐2 method to synthesize Ni/NG‐1′, and to obtain Ni/NG‐2′ in a similar way (see method in Supporting Information), resulting in no significant ECL emission peaks (Figure 3J) or several concurrent peaks (Figure 3K). Finally, the predicted catalytic mechanism schematic diagram is shown in Figure 3A.

## 5 Co‐Reactant ROS Pathway and Other Pathways in Ru(bpy)_3_^2+^ ECL

**FIGURE 4:**
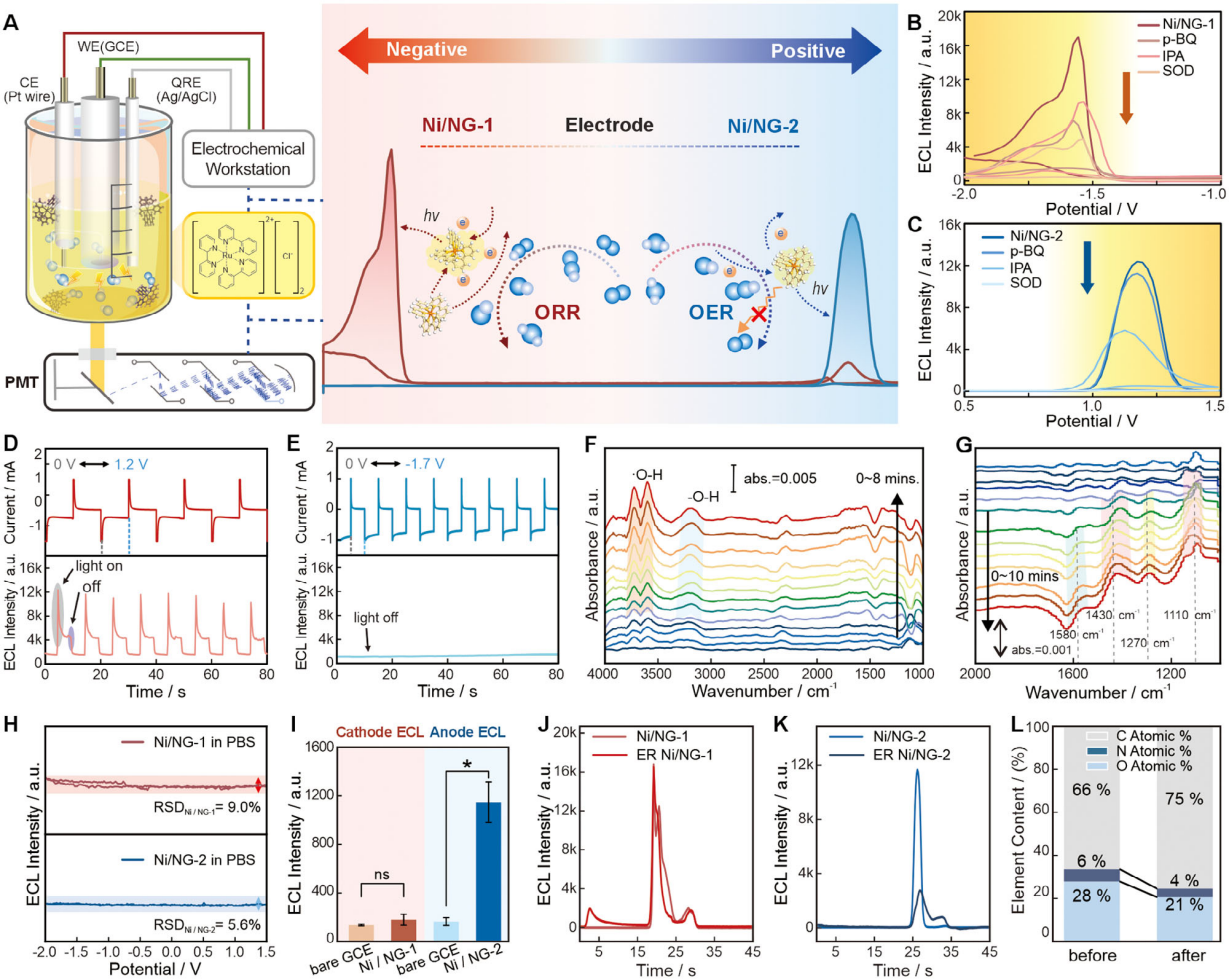
ROS products of ORR/OER reactions and other co‐reactant pathways. (A) Mechanistic illustration of ROS promoted Ru(bpy)_3_^2+^ ECL system. ECL intensity after addition of radical scavengers of (B) Ni/NG‐1 and (C) Ni/NG‐2. Chronoamperometry of Ni/NG‐1 at (D) ≈0–1.2 V and Ni/NG‐2 at (E) ≈−1.7–0 V. In situ FT‐IR spectra of (F) Ni/NG‐1 and (G) Ni/NG‐2. (H) Ni/NGs’ ECL in 0.1 M PBS. (I) Ru(bpy)_3_^2+^ ECL in an acetonitrile solution of Ni/NG and GCE. Comparison of ECL in Ru(bpy)_3_^2+^ before and after electroreduction for (J) Ni/NG‐1 and (K) Ni/NG‐2. (L) The C, N, and O elemental contents of Ni/NG‐2 before and after ECL test determined by XPS.

Building on the varying activation capacities of ORR/OER, the relationship between ROS generation and Ru(bpy)_3_^2+^ ECL performance was further investigated (Figure 4A). ROS quenchers were introduced into the system, including *p*‐benzoquinone (*p*‐BQ, •O_2_H), isopropanol (IPA, •OH), and SOD (•OH). The addition of these quenchers significantly decreased the luminescence in the Ni/NG‐1 group (Figure 4B), confirming the important role of •O_2_H and •OH. In contrast, for Ni/NG‐2 (Figure 4C), reduced luminescence occurred mainly with IPA and SOD groups, indicating that •OH is the main co‐reactant responsible for anodic ECL. Chronoamperometry and CV were compared to explore the progression of the ORR/OER (Figure 4D,E; Figures S14 and S15). When the pulse potential is −1.7 and 0 V, Ni/NG‐1 has a cathodic ECL at −1.7 V, which can be speculated that its weaker ORR catalytic ability prevents complete reaction, regardless of slow CV scans or direct voltage application [44]. Interestingly, at +1.2 V and 0 V, Ni/NG‐1 shows ECL at +1.2 V, suggesting that a high and direct potential causes Ni/NG‐1 to form intermediate products (e.g., •O_2_H) in situ during the OER, which contributes to luminescence. In contrast, cyclic scans revealed that Ni/NG‐1 initiated OER reactions at approximately −1.0 V, resulting in short‐lived •OH and H_2_O_2_ species that decompose and reduce their effectiveness (Figure 4D; Figure S15B). While Ni/NG‐2 shows minimal current between −1.7 and 0 V (Figure 4E), which is caused by its primary 4e^−^ ORR pathway leading to direct generation of H_2_O. The weak OER process allows it to directly produce massive ROS at +1.2 V (onset potential at 0.2 V), leading to favorable luminescence (Figure S15).

We used in situ FTIR spectroscopy to detect various oxygen‐containing intermediates formed during the ORR process [45, 46, 47]. Figure S16 shows the absence of distinct peaks in N_2_‐saturated conditions, indicating that all peaks belong to intermediates and products of the ORR. Figure S17 shows the voltage dependence of Ni/NGs. In Figure 4F, the prominent peak of Ni/NG‐1 near 3735 cm^−1^ points to the telescopic vibration of the free hydroxyl group. The lower vibrational frequency of the peak at 3215 cm^−1^ proves that the hydroxyl groups involve hydrogen bonds, and abundant ROS are produced during the process. For Ni/NG‐2, peaks of the O─O stretching mode around 1430 and 1110 cm^−1^ (Figure 4G) and the occurring peak at 1270 cm^−1^ slowly with time, which indicates the bending mode vibration of O_2_H*(abs) on the catalyst surface. Electron transfer from NiNPs to the O_2_ active site weakens the O─O bond, as evidenced by a decrease in the wave number at 462 s, which facilitates the 4e^−^‐transfer mechanism of ORR. After 600 s, fresh O_2_ molecules were adsorbed, restoring the wave number. In addition to these ORR intermediate peaks, an H─O─H bending mode appears near 1580 cm^−1^ and the broad hump near 3750 cm^−1^ of O─H further confirms the formation of H_2_O (Figure S18) [39]. This elaborates the relationship between determined ORR/OER strength and potential‐resolved luminescence in Ru(bpy)_3_^2+^ system (Figure 4A).

What is the other possible pathway for Ni/NGs to promote ECL emission? Ni/NG catalysts alone do not emit ECL in PBS (Figure 4H), indicating they cannot act as ECL emitters on their own. Ultraviolet scanning (Figure S19) shows a predominant absorption wavelength around 300 nm, confirming no energy resonance transfer between Ni/NGs and Ru(bpy)_3_^2+^ (454 nm). The co‐reactant properties of Ni/NGs were also measured by testing the ECL in an acetonitrile solution dissolving Ru(bpy)_3_^2+^ powder, with H_2_O and O_2_ removed (see method in Supporting Information) (Figure 4I). Ni/NG‐1's cathodic ECL is similar to that of GCE, while Ni/NG‐2 still enhanced anodic ECL (Figure 4J,K), implying its co‐reactant properties. XPS analysis was consumed to verify the changes in O and N content of Ni/NG before and after 1 h of ECL reaction (Figure 4L; Figure S20; Table S2), revealing a noticeable reduction of N and O in Ni/NG‐2 group. In all, Ni/NG‐2 promotes the luminescence through a synergistic reaction pathway by depleting its groups such as hydroxyl and amino groups, like the reaction pathway of TPrA, and the ROS pathway. Diverse types of radical species containing both amino and hydroxyl radicals are formed, explaining the less pronounced reduction current in Ru(bpy)_3_^2+^ during the reverse scan than in PBS (Figure 2K).

In summary, the cathodic CRA, Ni/NG‐1, primarily stems from its 2e^−^ transfer pathway and weak ORR catalytic activity, causing the continuously produced ROS to interact with Ru(bpy)_3_^+^. And anodic CRA Ni/NG‐2 stems from its •OH generation during the OER. The luminous mechanism and equations are shown in equation (Figure S21).

## 6 Practical Application in Immunosensors

**FIGURE 5:**
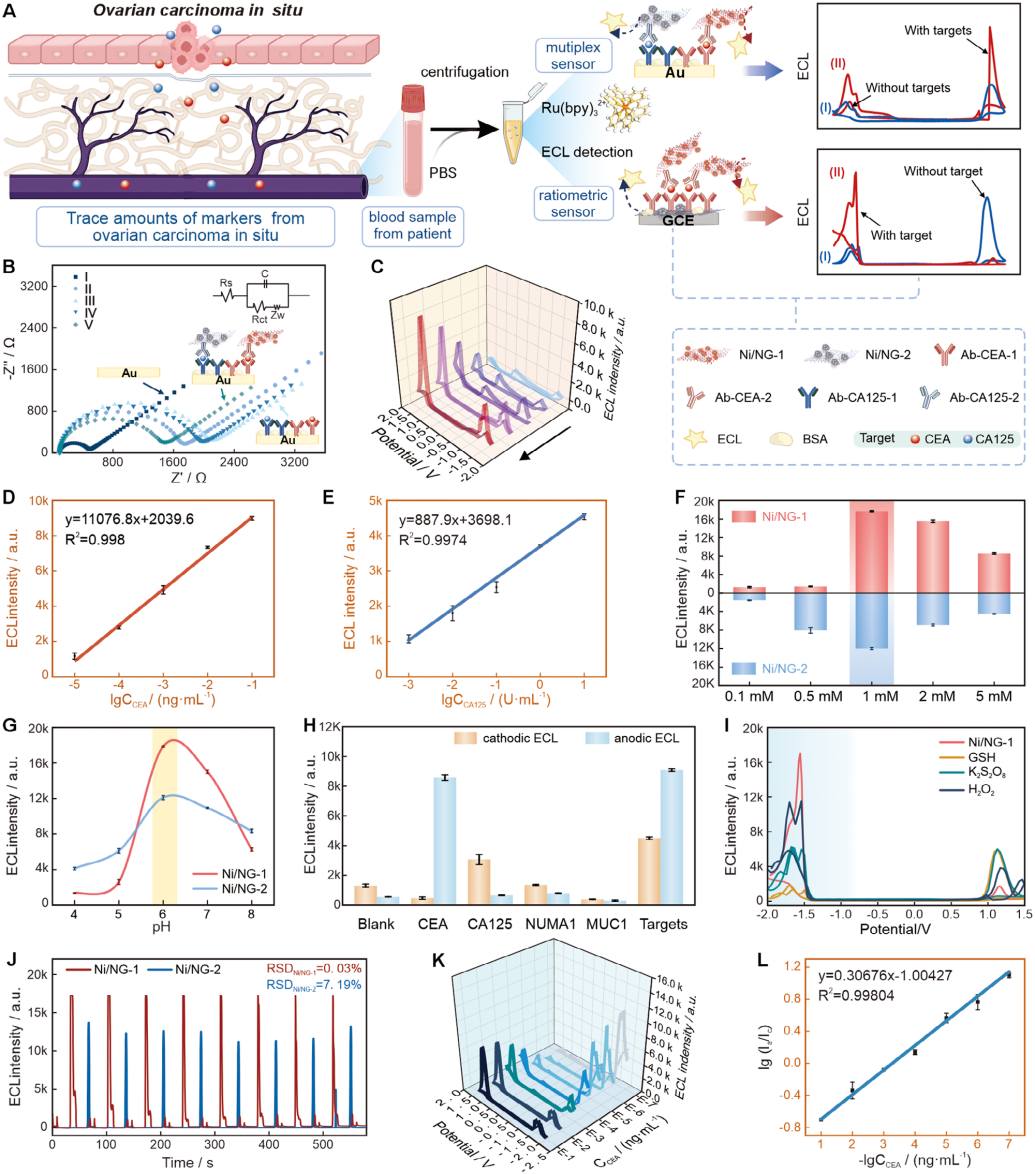
Immunosensor construction and detection performance. (A) Schematic illustration of the “sandwich” type strategy for multiplex tumor markers ECL immunosensor and the ratiometric ECL immunosensor. (B) EIS of multi‐marker ECL biosensor, from ≈0–4000 Ω of (I) bare Au, (II) anti‐mix antibody‐1/Au, (III) mix Antigen/BSA/anti‐mix antibody‐1/Au, (IV) Ni/NG‐1‐antibody‐CEA‐2/mix antigen/BSA/anti‐mix antibody‐1/Au, and (V) Ni/NG‐2‐antibody‐CA125‐2/Ni/NG‐1‐antibody‐CEA‐2/mix Antigen/BSA/anti‐mix antibody‐1/Au. EIS was measured in 0.1 mol/L of KCl containing 5.0 mmol/L of K_3_[Fe(CN)_6_]/K_4_[Fe(CN)_6_] (1:1). *R*_s_, solution resistance; *C*_dl_, double‐layer capacitance; *R*_ct_, charge transfer resistance; *Z*_w_: Warburg impedance. (C) ECL response for multiplex tumor markers ECL immunosensor. (D,E) Calibration plot of the ECL intensity with different concentrations of CEA and CA125. (F) The optimal Ru(bpy)_3_^2+^ concentration. (G) The optimal pH conditions. (H) The selection of the proposed multi‐marker ECL biosensor. (I) ECL comparison of Ni/NG‐1 with other different cathodic co‐reactants. (J) Stability ECL of Ni/NG. (K) ECL response for ratiometric ECL immunosensor and (L) its calibration plot of the ECL intensity with different concentrations of CEA.

Utilizing pure and stable ECL signals, these two Ni‐CRAs were employed to construct two types of immunosensing platforms: A multiplex sensor and a single‐marker detection ratiometric sensor (Figure 5A). For the multiplex sensor, CEA and CA125, two essential biomarkers for ovarian cancer, were selected as the model (Figure S22; see method in Supporting Information). Electrochemical impedance spectroscopy (EIS) was performed to track the sensor preparation process and confirm the successful construction of this immunosensor (Figure 5B).

The properties of the sensors are illustrated in Figure 5C–G. The ECL signal increases gradually with the addition of the antigen, showing good linearity within the ranges of ≈10^−5^–0.1 ng mL^−1^ for CEA and ≈10^−3^–10 U mL^−1^ for CA125 (Figure 5C), with detection limits of 6.57 × 10^−10^ g mL^−1^ and 7.20 × 10^−5^ U mL^−1^, respectively (*S*/*N* = 3) (Figure 5D,E). Prior to experiments, the detection conditions for the two antigen‐antibody reactions (Figure 5F,G), encompassing the Ru(bpy)_3_^2+^ concentration (1 mM) and the pH value of the solution (pH = 6), were tuned to the optimum [48]. The selectivity of the system was also evaluated by introducing various heterologous antigens into the detection process, thereby confirming its high specificity and reliability (Figure 5H). The immunosensor also exhibited high recovery rates (90.01% to 109.64%) in real human samples, which closely align with the commercialized ECLIA method (Table S3).

Notably, despite the traditional rarity of cathodic CRAs, Ni/NG‐1 exhibited excellent cathodic ECL performance (Figure 5I), and the Ni/NG catalysts demonstrated superb, stable, and unaffected ECL across a range of potentials (Figure 5J). These outstanding CRAs have inspired further exploration of new CRA combinations, with LSV demonstrating the potential of other non‐precious metals as CRAs (Figure S23). For the single‐marker detection ratiometric sensor (Figure S24), extended detection ranges (Figure 5K,L), excellent selectivity, and reliable real sample testing were also achieved (Figure S25; Table S4) [49]. This application further verified the practical value of this ECL immunosaasy system.

## 7 Conclusion

In summary, facet‐tunable Ni/NG catalysts were systematically designed to optimize ORR/OER catalytic capabilities and tailor the generation of ROS, a key determinant of Ru(bpy)_3_^2+^ ECL emission and the switch between cathodic/anodic ECL. Ni/NG‐1, with limited ORR activity and slower kinetics, generates substantial ROS, resulting in strong and stable cathodic ECL, while its enhanced OER activity inhibits anodic ECL by reducing oxygen byproducts on the catalyst surface. In contrast, Ni/NG‐2, with weak OER activity, promotes strong anodic ECL, and its 4^−^electron ORR pathway directly to H_2_O eliminates cathodic ECL. Theoretical calculations revealed that the Ni(1 1 0) facet has the highest Δ*G* for the fourth ORR step, while the strong •OH adsorption on Ni hinders H_2_O* desorption, corroborating experimental results. This study represents the first investigation into the surface‐dependent ROS formation kinetics of the specific radical species during the ORR, and builds a bridge between ROS and the Ru(bpy)_3_^2+^ ECL system. This approach not only offers a promising strategy for predicting the ECL performance by taking full use of these CRAs with weak ORR/OER catalytic abilities through DFT, thereby advancing the design of non‐precious metal CRAs, but also innovatively applies the NPs‐ECL‐ROS system for practical detection, enabling richer information acquisition in clinical immunoassays.

O_2_ molecules undergo ORR at the cathode, while H_2_O molecules undergo OER at the anode on different catalyst facets. Ni/NG‐1 exhibits high OER but low ORR activity, generating radicals that react with Ru(bpy)_3_^2^⁺ to enhance cathodic ECL. In contrast, Ni/NG‐2 shows high ORR but low OER activity, thus promoting anodic ECL through a similar radical‐assisted pathway.

## Conflicts of Interest

The authors declare no conflicts of interest.

## Ethics Statement

This study was approved by the Ethics Committee of Chongqing Medical University (Approval No. 2023011). Written informed consent was obtained from all participants prior to their inclusion in the study. All procedures involving human participants were conducted in accordance with the Declaration of Helsinki.

## Supporting information




**Supplementary File 1**: exp270086‐sup‐0001‐SuppMat.docx.

## Data Availability

The data that support the findings of this study are available from the corresponding author upon reasonable request.
