# Supplementary material for: Switching Cathodic/Anodic Electrochemiluminescence of Ru(bpy)3 2+ Precisely via Homogeneous Nickel Nanoparticles Crystal Facets Sites Modulated ORR/OER
Source: Exploration (Beijing). 2025 Aug 30;5(5):20250036. doi: 10.1002/EXP.20250036 (PMC12561308; doi:10.1002/EXP.20250036)
Supplement: Supplementary file 1 — exp270086‐sup‐0001‐SuppMat.docx. [file EXP2-5-20250036-s001.docx]

Supporting Information

Switching Cathodic/Anodic Electrochemiluminescence of Ru(bpy)_3_^2+^ Precisely via Homologous Nickel Nanoparticles Crystal Facets Sites Modulated ORR/OER

Experimental Section

Apparatus and Characterizations
The ECL measurements were performed on a MPI-multifunctional electrochemiluminescence analyzer (Xi’an Remex Analytical Instrument Ltd. Co., China). The three-electrode ECL cell was consisted of a modified glassy carbon as the working electrode (φ = 3 mm), an Ag/AgCl (KCl saturated) electrode as the reference, and a platinum wire as the counter electrode. Gold electrodes were used in the immunosensor to attach antibodies. The photomultiplier tube (PMT) was biased at 600 V, and the scan voltage was from 1.5 to -2 V with the scan rate of 100 mV s^-1^. Ultraviolet−visible light (UV-vis) absorption spectra and fluorescence spectra were obtained with a spectrophotometer (Model UV2450, Shimadzu, Japan) and a spectrophotometer (Model F-7000, Hitachi, Japan), respectively. X-ray photoelectron spectroscopy (XPS) characterizations were measured by a VG Multilab 2000X instrument (Thermal Electron, USA). Fourier transform infrared spectra (FT-IR) were observed on a FT-IR spectrometer (ALPHA, Bruker). Transmission electron microscope (TEM) images were obtained using a JEM-2010 transmission electron microscopy (JEOL, Japan). Cyclic voltammograms (CVs) and electrochemical impedance spectroscopy (EIS) were carried out on an electrochemical workstation (chinstruments, Shanghai). CVs were recorded in a potential range between −0.2 V and + 0.6 V at a scan rate of 100 mV/s in a solution of 5 mM K_3_[Fe(CN)_6_]/K_4_[Fe(CN)_6_] containing 0.1 M KCl. EIS measurements were performed by applying a voltage of 5 mV amplitude in 0.01 Hz to 106 Hz frequency range.

In situ attenuated total reflection Fourier transformed infra-red (ATR-FTIR) spectroscopy. In situ electrochemical FTIR spectroscopic studies were performed using a purged VERTEX FT-IR spectrometer equipped with the A530/P accessory and a mid-band MCT detector. A CaF_2_ hemispherical window was used with the working electrode placed 1 mm above the window for the FTIR study. The measurement parameters were 0.09 cm^-1^ resolution and at different voltages. This setup enabled the detection of ORR intermediate formation and change of adsorption of various intermediates on the electrode surface and within the thin-layer electrolyte.

Materials and Methods

Nickel nitrate (Ni(NO_3_)_2_), Cobalt chloride (CoCl_2_), Chloride hexahydrate iron (FeCl_3_·6H_2_O), copper nitrate (Cu(NO_3_)_2_), pyrrole and sodium citrate were obtained from Aladdin Reagent Company. Tris(2,2’-bipyridine) ruthenium(II) (Ru(bpy)_3_^2+^) was acquired from Suna Tech Inc. Natural graphite powder (325 mesh) was purchased from Nanjing XFNANO Materials Tech Co.,Ltd. Ethanol, isopropanol, ethylene glycol, hydrazine hydrate, potassium persulfate and hydrogen peroxide were obtained from Sinopharm Chemical Reagent Co. Ltd. N-Hydroxy succinimide (NHS, GR) and 1-ethyl-3-(3-di-methylaminopropyl) carbodiimide hydrochloride (EDC, GR) were acquired from Shanghai Medpep Co., Ltd. (Shanghai, China). Bovine serum albumin (BSA, 96−99%, GR) was purchased from Biss Inc. (Beijing, China). Carcinoembryonic antigen (CEA) and antibodies, Sugar chain antigen (CA125) and antibodies, Nuclear Mitotic Apparatus protein 1 (NUMA1) and mucin (MUC1) were supplied by Cloud Clone Corp.Wuhan (Wuhan, China). Human serum sample were provided by Department of Pathology, Guizhou Provincial People's Hospital. Phosphate buffer solution (PBS, 0.1M pH 7.4) containing KH_2_PO_4_ and Na_2_HPO_4_ in an appropriate ratio with KCl (0.1 M) was used for ECL detection. All other chemicals were of analytical grade and used as received without further purification. All the aqueous solutions were freshly prepared and diluted with ultrapure water (≥18MΩ, Milli-Q, Millipore).

Experimental Design

*Preparation of GO.* GO was synthesized by the oxidation of natural graphite powder employing Hummers' method,^[1]^ with a few modifications. Briefly, graphite powder (3.0 g) was added to concentrated H_2_SO_4_ (70 mL) and vigorously agitated in an ice bath. Then, 9.0 g of KMnO_4_ was gently added to maintain the suspension temperature below 20 ℃. The reaction system was successively transferred to a 40 ℃ oil bath and aggressively agitated for approximately 30 minutes. After that, 150 mL of water was added, and the solution was agitated for 15 minutes at 95 ℃. A further 500 mL of water was added, followed by a steady addition of 15 mL of H_2_O_2_ (30%), which changed the color of the solution from dark brown to yellow. To remove metal ions, the mixture was filtered and washed with a 250 mL of 1:10 HCl aqueous solution. The resultant solid was dried in air and diluted to 600 mL to create an aqueous graphite oxide aqueous dispersion. Finally, it was dialysis-purified for one week using a dialysis membrane (Beijing Chemical Reagent Co., China) with a molecular weight cut-off 8000−14000 g·mol^−1^ to eliminate the residual metal species. The resulting graphite oxide aqueous dispersion was then diluted to 1.2 L, agitated overnight, and sonicated for 30 minutes to exfoliate it to GO. The GO dispersion was then centrifuged at 3000 rpm for 40 minutes to remove the unexfoliated graphite. 4 mg graphene oxide was dissolved in 2 mL of ultrapure water and the resulting 2 mg mL^-1^ graphene oxide dispersion.

*Preparation of Ni/NG-1.* The composite of N-doped graphene/nickel nanoparticles (denoted as Ni/NG-1) was prepared using a simple synthesis method. 2.5 μL of 3 mM Ni(NO_3_)_2_ solution was added to 2 mL of graphene oxide dispersion under stirring, and the above solution was sonicated for 10 minutes and then stirred for 2 hours at room temperature. The process was repeated 3 times. 25 μL of 0.01 M sodium citrate was quickly added to the solution, stirred for 15 minutes and sonicated for 5 minutes. 4 μL of pyrrole (analytically pure) was added dropwise to the solution and stirred for 30 minutes; finally, it was centrifuged at 12000 rpm for 2 hours. The precipitate was taken and resuspended.

*Preparation of Ni/NG-2.* Ni/NG-2 was prepared by a simple three-step method. In the first step, 0.2 mL of pyrrole (analytically pure),^[2]^ 0.5 mL of aqueous nickel nitrate (0.3 M), 1 mL of ethylene glycol (analytically pure), and 0.125 mL of hydrazine hydrate (analytically pure) were added to 2 mL of graphene oxide dispersion step by step.^[3]^ The mixture was then loaded into a stainless steel autoclave lined with polytetrafluoroethylene and heated in an oil bath at 120℃ for 12 hours. In the second step, the resulting mixture was loaded into a centrifuge tube and centrifuged at 10000 prm for 10 minutes, the supernatant was removed, and the sediment was dispersed with 4 mL of ultrapure water, and the process was repeated twice. In the third step, the suspension was further dispersed using a homogenizer for 1 hour and sonicated for 20 minutes, resulting in a Ni/NG-2 sample.

*Preparation of Ni/NG-1’.* 2 mL of graphene oxide dispersion (2 mg mL^-1^), 2.5 μL of nickel nitrate solution (3 mM), 25 μL of sodium citrate (0.01 M), and 4 μL of pyrrole were mixed. The above solutions were loaded into a stainless steel autoclave lined with polytetrafluoroethylene, and the material Ni/NG-1' was synthesized in an oil bath at 120℃ for 12 hours.

*Preparation of Ni/NG-2’.* To 2 mL of graphene oxide (GO) dispersion was added 0.5 mL of nickel nitrate (0.3 M), and the mixed solution was sonicated for 10 minutes, followed by stirring at room temperature for 2 hours. The process was repeated three times. The process was repeated three times and again sonicated for 10 minutes. 0.2 mL of pyrrole (2 mg/mL) was added to the above solution and stirred for 20 minutes. 1 mL of ethylene glycol, 0.125 mL of hydrazine hydrate was added to the above solution, stirred for 30 minutes, and sonicated for 30 minutes. Subsequently, the resulting mixture was loaded into a centrifuge tube and centrifuged for 2 hours at 12000 prm, and the supernatant was removed. The precipitate was taken and finally the resulting Ni/NP-2' sample was resuspended in ultrapure water.

*Preparation of Ab_2_ bioconjugates.* Initially, the as-prepared Ni/NG-1 was sonicated for 30 minutes before used. After that, 120 μL of the 2 mg mL^-1^ Ni/NG-1 were dispersed in newly prepared EDC (0.01 mol L^-1^)/NHS (0.002 mol L^-1^) solution.

To the above solution, 120 mL of antibody-CEA-2 solution was added, placed in a 1.5 mL Eppendorf tube, and incubated for 10 hours at 4℃ under constant temperature and shaking conditions. After centrifugation (5000 rpm, 15 minutes), it was washed with PBS solution to remove unbound antibody-CEA-2. 120 mL of 1% BSA solution was added to block the non-specific sites and oscillated at 4 ℃ for 2 hours. The mixture was then centrifugated at 5000 rpm for 15 minutes. Finally, the obtained Ni/NG-1-antibody-CEA-2 was redistributed into 120 mL of pH 7.4 PBS solution and stored at 4 ℃ until use. The Ni/NG-2-antibody-CA125-2 bioconjugates are treated in the same way as described above.

*Fabrication procedure for the multi-marker ECL biosensor.* The fabrication process of the ratiometric ECL immunosensor was shown in Figure 5a, Figure S22. Prior to modification, the bare Au electrode was cleaned by physical polishing and chemical/electrochemical washing and checked in the reversible electrode reaction of the Fe(CN)_6_^3-^/Fe(CN)_6_^4-^ pair. Then 3 μL of mixed capture antibody was dropped onto the cleaned Au electrode and dried at room temperature. After washing with PBS, 5 μL of 1.0 wt% BSA was dipped onto the modified electrode to block the nonspecific adsorption sites. Subsequently, 5 μL of mixed antigen with different concentrations (CEA 10^-1^, 10^-2^, 10^-3^, 10^-4^, 10^-5^ ng mL^-1^, CA125 10，1，10^-1^, 10^-2^, 10^-3^ U mL^-1^) was added on the decorated electrode and incubated for 1 hour at 37 ℃. After washing with PBS, 5 μL mixed Ab_2_ bio-conjugate was incubated on the modified electrode for 1 hour at 37℃ to construct multi-marker ECL immunosensor.

*Fabrication procedure for the ratiometric ECL biosensor.* The fabrication process of the ratiometric ECL immunosensor was shown in Figure 5a, Figure S24. Prior to modification, the bare GCE was cleaned by physical polishing and chemical/electrochemical washing and checked in the reversible electrode reaction of the Fe(CN)_6_^3-^/Fe(CN)_6_^4-^ pair. Then 5 μL of 2 mg mL^-1^ Ni/NG-2 was dropped onto the cleaned GCE and dried at room temperature. Subsequently, 5 μL of EDC/NHS solution was used to activate the carboxyl group and amino group of Ni/NG-2 for 30 minutes at 37℃. After washing with ultrapure water, 5 μL of 20 μg mL^-1^ capture antibody (Anti-CEA-1) was incubated on the modified electrode for 1 hour at 37℃. After removing excess reagent, 5 μL of 1.0 wt% BSA was dipped onto the modified electrode to block the nonspecific adsorption sites. Subsequently, 5 μL of CEA with different concentrations (10^-1^, 10^-2^, 10^-3^, 10^-4^, 10^-5^, 10^-6^, 10^-7^ ng mL^-1^) was added on the decorated electrode and incubated for 1 hour at 37℃. After washing with ultrapure water, 5 μL CEA Ab_2_ bio-conjugate was incubated on the modified electrode for 1 hour at 37℃ to construct ratiometric ECL immunosensor.

Application of the ratiometric sensing platform for bioanalysis.

CEA and CA125 was detected on a double antibody sandwich modified electrode in a solution of 100 mM pH 6.0 PBS containing 1 mM Ru(bpy)_3_^2+^ at room temperature using an ECL method with 600 V bias and a potential scan spanning from +1.5 V to -2 V.

Electrochemical Measurement.

Cyclic Voltammetry (CV). CV was performed using a computer-controlled potentiostat (CHI660D) in a standard three-electrode cell using Ag/AgCl (KCl saturated) electrode, a platinum wire as the counter electrode and a glassy carbon electrode (GCE) as the working electrode. 5 μL of Ni/NG suspension was dropped onto a GCE with a diameter of 3 mm. The three electrodes were put into 0.1 M KOH solution. A flow of O_2_ or N_2_ was maintained for 40 minutes to achieve O_2_-saturated or O_2_-free. The working electrode was cycled at least 5 times in the potential range from -2.0 to 1.5 V at a scanning rate of 100 mV s^-1^. The electrochemical double-layer capacitance (C_dl_) was measured by typical CV at different scanning rates (20, 40, 60, 80,100 and 120 mV s^−1^) at -0.35 to -0.45 V compared with Ag/AgCl. Then, the electrochemical surface area (ECSA) was calculated according to the C_dl._ values.

Rotating Ring–disk Electrode(RRDE). A three–electrode system was used, with an Ag/AgCl electrode as the reference electrode, a platinum wire as the counter electrode, and a catalyst–modified rotating ring–disk electrode (RRDE) as the working electrode. The catalysts were prepared as follows: 10 μL Ni/NG-1 was dispersed in 8 μL of 5 wt.% Nafion solution. The mixture was sonicated for approximately 15 minutes to obtain a homogeneous ink.

Subsequently, 10 μL of the prepared ink was dropped onto a polished RRDE (d = 4 mm) and dried at room temperature. Potential reference reversible hydrogen electrode (RHE): E (RHE) = E (Ag/AgCl) + 0.059 × pH + 0.197.

For the RRDE, the electron transfer number (n) is determined by the current density ratio of the ring/disk electrode, as determined by equation (1):

$\text{n = }\frac{\text{4 ×}\text{ I}_{\text{d}}}{\text{I}_{\text{d + }{\text{I}_{\text{r}}}/\text{N}}}$ (1)

and the selectivity of H_2_O_2_ on RRDE is calculated according to equation (2):

$\text{H}\text{2}\text{O}\text{2}\text{ }\left( \text{\%} \right)\text{= }\frac{\text{200 ×}\text{ I}_{\text{r}}}{\left( \text{N × }\text{I}_{\text{d}} \right)\text{ + }\text{I}_{\text{r}}}$ (2)

where I_r_ is the ring electrode current, I_d_ is the disc electrode current, and N is the ring electrode collection efficiency (0.37). The ring current was recorded by maintaining the Pt ring at 1.2 V (with respect to RHE) throughout the test.

ECL characterization

ECL measurements were performed using an ultramicroscopic luminometer (BPCL–GP21–Q) in combination with a homemade CHI 700E instrument. A GCE was used as the working electrode, and Pt and saturated Ag/AgCl electrodes were used as the counter and reference electrodes, respectively. The catalyst solution (5 μL) was then added dropwise to the GCE surface and dried at 37 ℃. The ECL emission was obtained in a 1 mM Ru(bpy)_3_^2+^ solution (pH adjusted to 6). The potential range was from +1.5 V to -2 V and the scan rate was 100 mV s^-1^. For ECL measurements of Ni/NG in different atmospheres, the test electrode is first purged in O_2_ (N_2_) atmosphere for 30 minutes until O_2_ (N_2_) saturation.

Electrochemical Impedance Spectroscopy (EIS)

These plots were recorded in a solution containing 5 mM K₃[Fe(CN)₆] solution containing 0.1 M KCl, with a frequency range from 500 Hz to 2 kHz. The impedance data were fitted to an improved Randles equivalent circuit, where Rs represents the solution resistance, C is the constant phase angle element, Rct is the charge-transfer resistance and Zw is the Warburg impedance.

Theoretical calculations

Density functional theory (DFT) Computational details

All DFT calculations were performed using Vienna ab initio simulation package (VASP) with projector augmented wave (PAW) pseudopotentials. The generalized gradient approximation(GGA) functional was used, and cutoff energy for the plane-wave basis set was set to 400 eV. The k-point sampling was obtained from the Monkhorst−Pack scheme with a 4 × 4 × 1 mesh. The computational models were established for Ni(2 0 0), Ni(1 1 1), and Ni(1 1 0) crystal face respectively. The crystal cell size was 1 × 1 and the boundary conditions in two directions were periodic to model a semi-infinite crystal surface. A vacuum of 20 Å was built for each slab model to avoid the interaction between adjacent layers and the Van der Waals force was taken into account during the calculation process. The convergence criteria of energy were set to 1×10^-5^ eV. The adsorption energies (E_ads_) of the reactants and products were calculated using Eq.: E_ads_ = E_surf+adsorbate_−E_surf_−E_adsorbate,_ where E_surf+adsorbate_ is the total energy of the surface-adsorbate system, E_surf_ is the total energy of the crystal surface and E_adsorbate_ is the total energy of the isolated, geometry optimized adsorbate. To obtain the energy of adsorbate, a 10×10×10Å mesh was built, with the same pseudopotentials and the convergence criteria of energy as above.^[4]^ The model of final adsorption was built and optimized, and the similar adsorption sites in the adsorption process are same in our simulation, which can exclude the influence of geometric factors of the adsorption process sites.


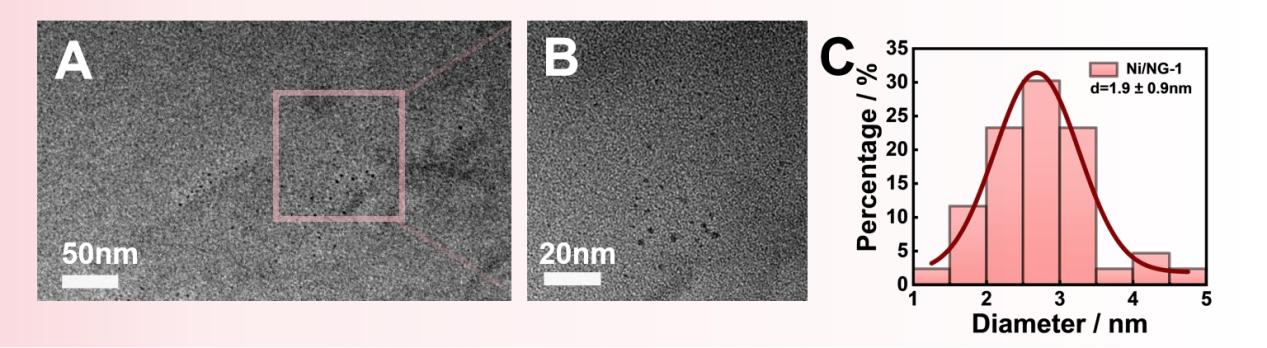


Figure S1. (A) TEM of Ni/NG-1. (B) The enlarged picture of (A). (C) The corresponding size histograms.


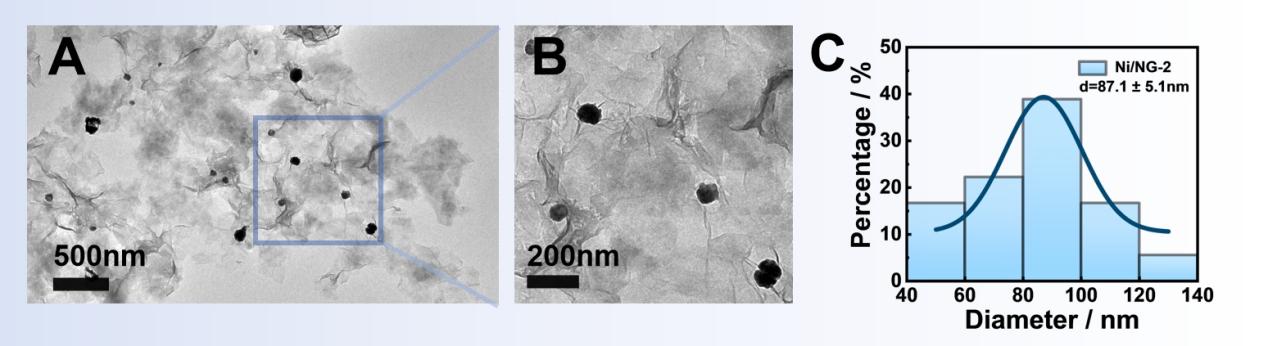


Figure S2. (A) TEM of Ni/NG-2. (B) The enlarged picture of (A). (C) The corresponding size histograms.

There is no free clusters formed outside the graphene oxide material. A significant difference exists in particle size between the cathodic and anodic co-reactant accelerator (CRA) Ni/NG. Under TEM at a scale of 20 nm and 200 nm respectively, particle size statistical analysis indicates that Ni/NG-1 exhibits NiNPs’ sizes around 1.9 nm, while Ni/NG-2 nanoparticle clusters have sizes around 87 nm.

Figure S3. EDS Mappings of Ni/NG-1.

The analysis of chemical component was conducted by EDS mapping. The presence of NiNPs on GO was further confirmed. It exhibits the characteristic peaks of C, O and Ni, consistent with chemical components of graphene and NiNPs.

Figure S4. EDS Mappings of Ni/NG-2.


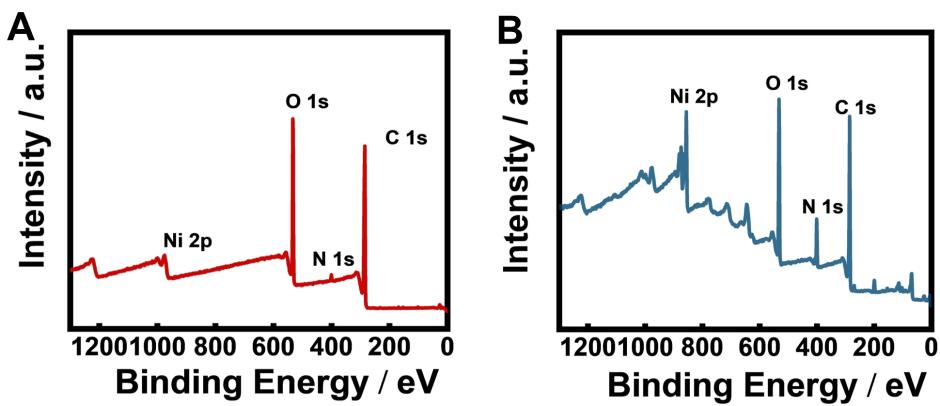


Figure S5. X-ray photoelectron spectroscopy (XPS) of Ni/NG-1(A) and Ni/NG-2(B).

The scanning spectra of the XPS measurements shows the presence of Ni, C, and N elements in the Ni/NG-1, Ni/NG-2 samples.


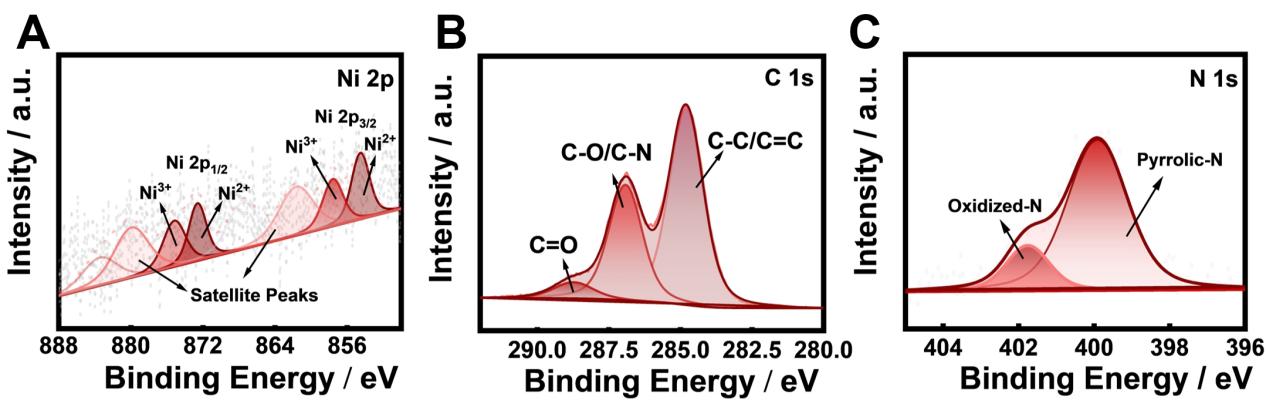


Figure S6. The scanning spectra of the XPS measurements shows the presence of Ni, C, and N elements in the Ni/NG-1.


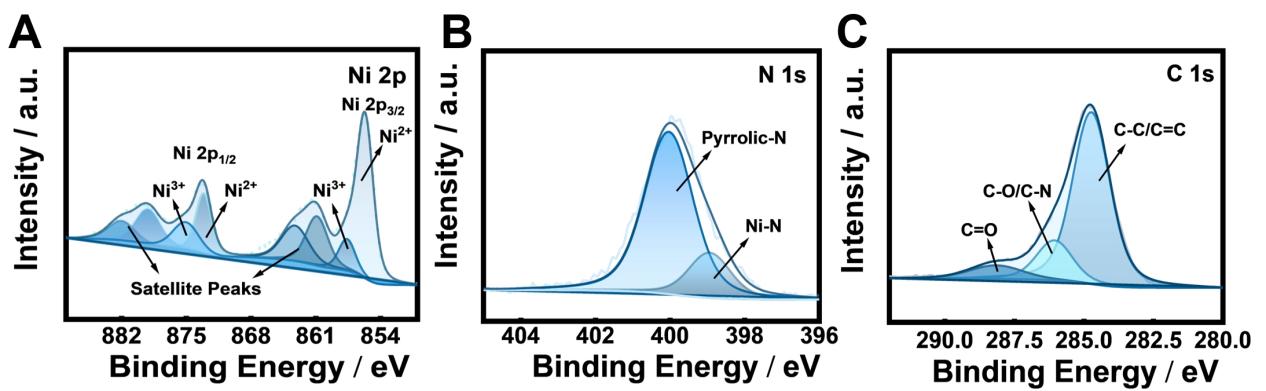


Figure S7. The scanning spectra of the XPS measurements shows the presence of Ni, C, and N elements in the Ni/NG-2.

Ni/NG-1 has an insignificant peak due to the very trace amount of Ni. In the C 1s pattern, the formation of C-N bonds with a binding energy of 287.25 uncovers that the N element is effectively doped into GO (Fig S6). For Ni/NG-2 (Fig S7), the appearance of Ni-N bonds in the 1s convolution peak of N indicates that N not only serves as an N source and a reducing agent, but also as a linking agent to connect the metal NPs. The high-resolution Ni 2p spectra exhibits binding energy peaks at 855.73 eV and 873.43 eV, which correspond to Ni 2p3/2 and Ni 2p1/2, respectively.

Additionally, there are satellite peaks with binding energy values of 860.83 eV and 882.08 eV, which correspond to the satellite peaks of Ni 2p3/2 and Ni 2p1/2, respectively. The Ni^2+^ peaks represent the presence of the Ni-N bonds, and the absence of Ni 0 peaks represents the absence of partial Ni metal presence residue, which may all form Ni-N bonds with N elements. The slight negative shift (~3.00 eV) of the Ni 2p3/2 spin-orbit doublet region (856 eV) with respect to the conventional reference position of metallic Ni (852.6 eV) suggests the presence of robust interactions between Ni and the GO framework.


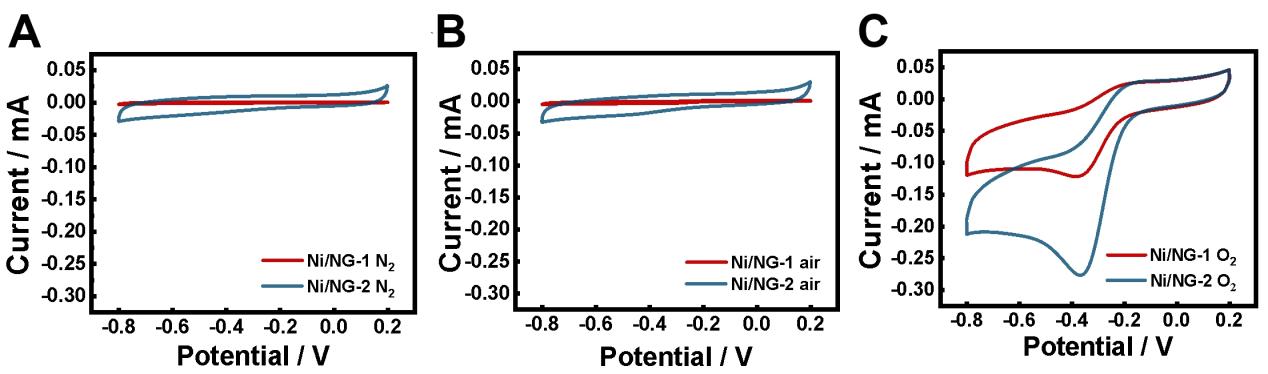


Figure S8. CV curves of Ni/NG in 0.1 M KOH in N_2_-saturated (A), air (B) and O_2_-saturated (C) atmospheres.


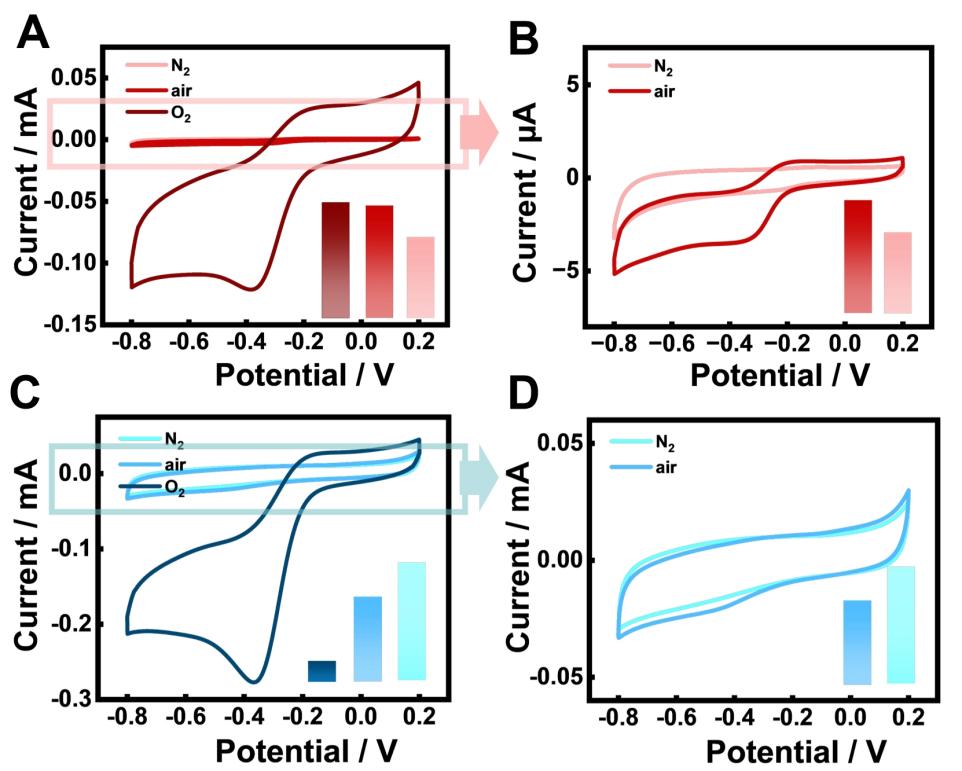


Figure S9. ORR curve and corresponding cathodic/ anodic ECL intensity histogram of Ni/NG-1 (A-B) and Ni/NG-2 (C-D).

Figure S10. The H_2_O_2_ yields of Ni/NG catalysts.

Figure S11. CV curves of Ni/NG in 1 mM Ru(bpy)_3_^2+^.


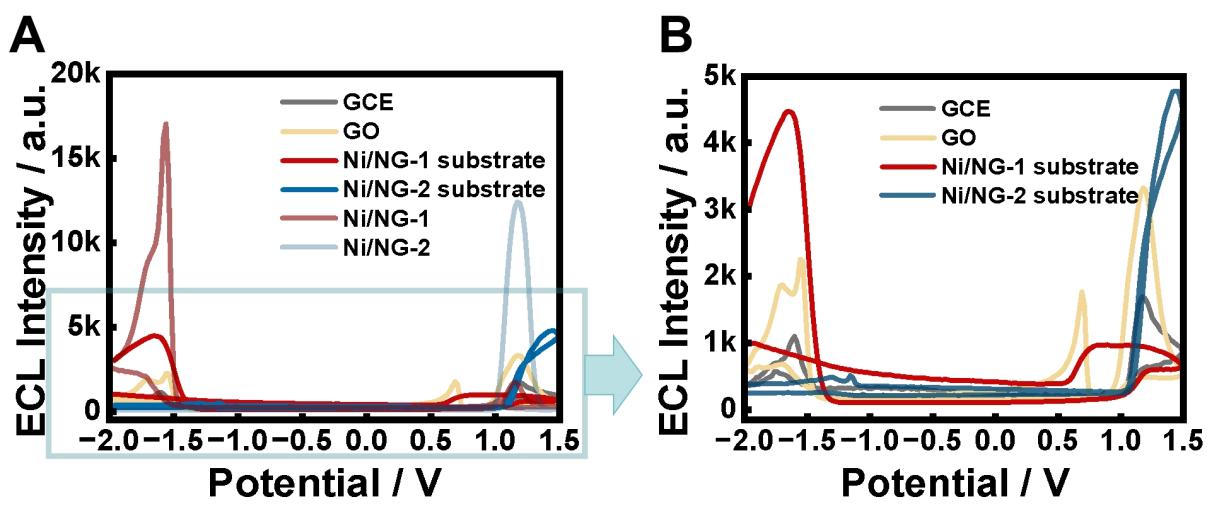


Figure S12. The ECL of Ni/NG’s substrates, Ni/NG catalysts, GCE and GO.


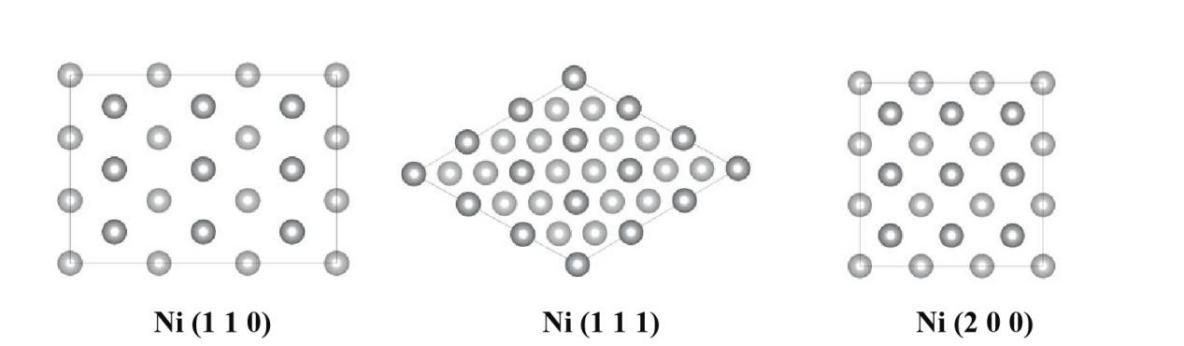


Figure S13. Surface model of Ni(1 1 0), Ni(1 1 1), and Ni(2 0 0).


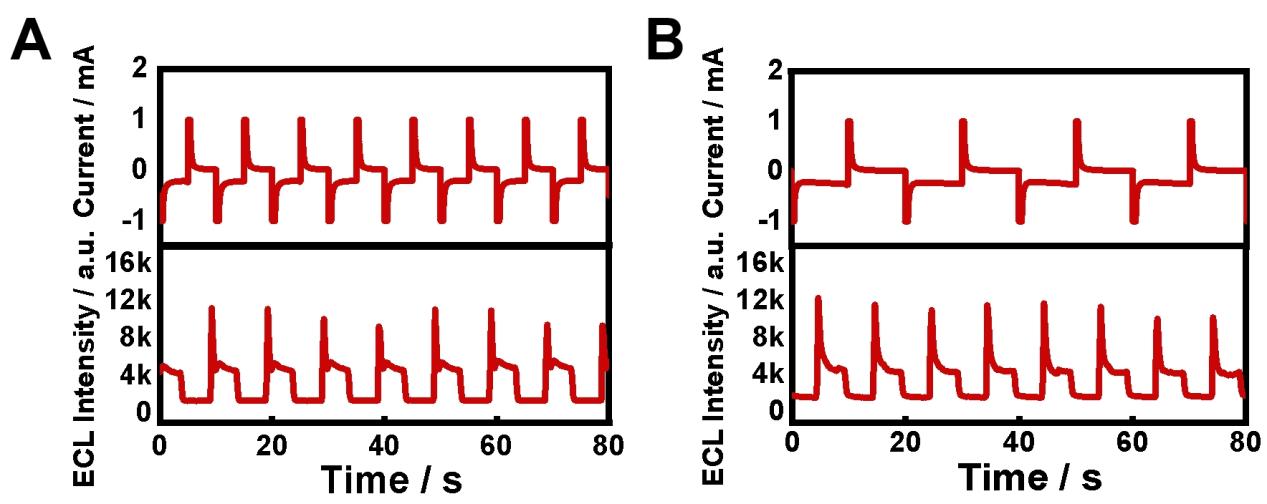


Figure S14. (A) Chronoamperometry of Ni/NG-1 between -1.7 V and 0 V. (B) Chronoamperometry of Ni/NG-1 between 0 V and 1.2 V.


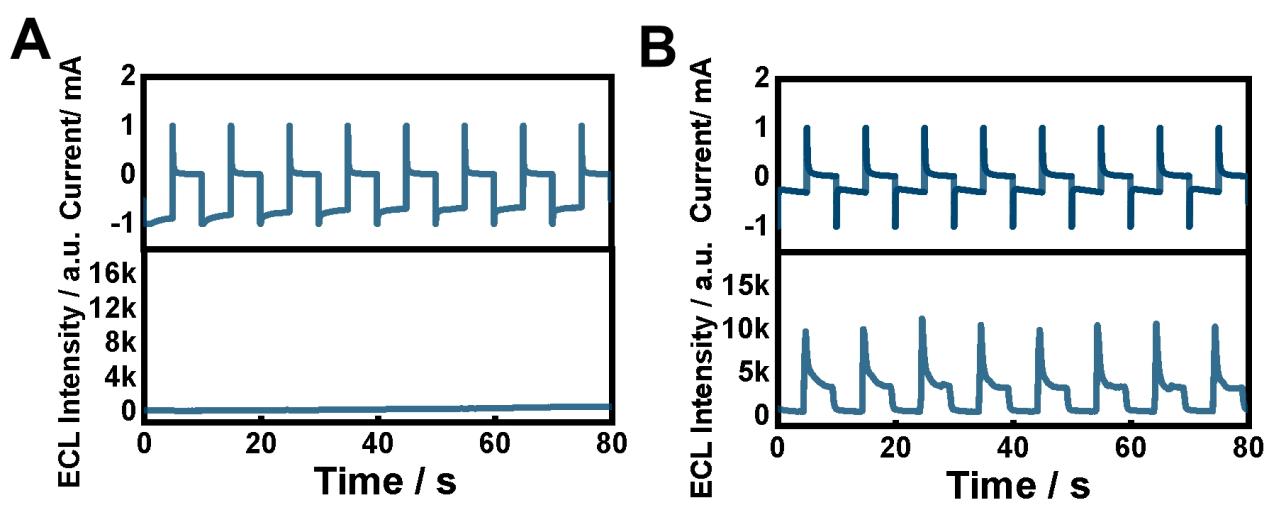


Figure S15. (A) Chronoamperometry of Ni/NG-2 between -1.7 V and 0 V. (B) Chronoamperometry of Ni/NG-2 between 0 V and 1.2 V.


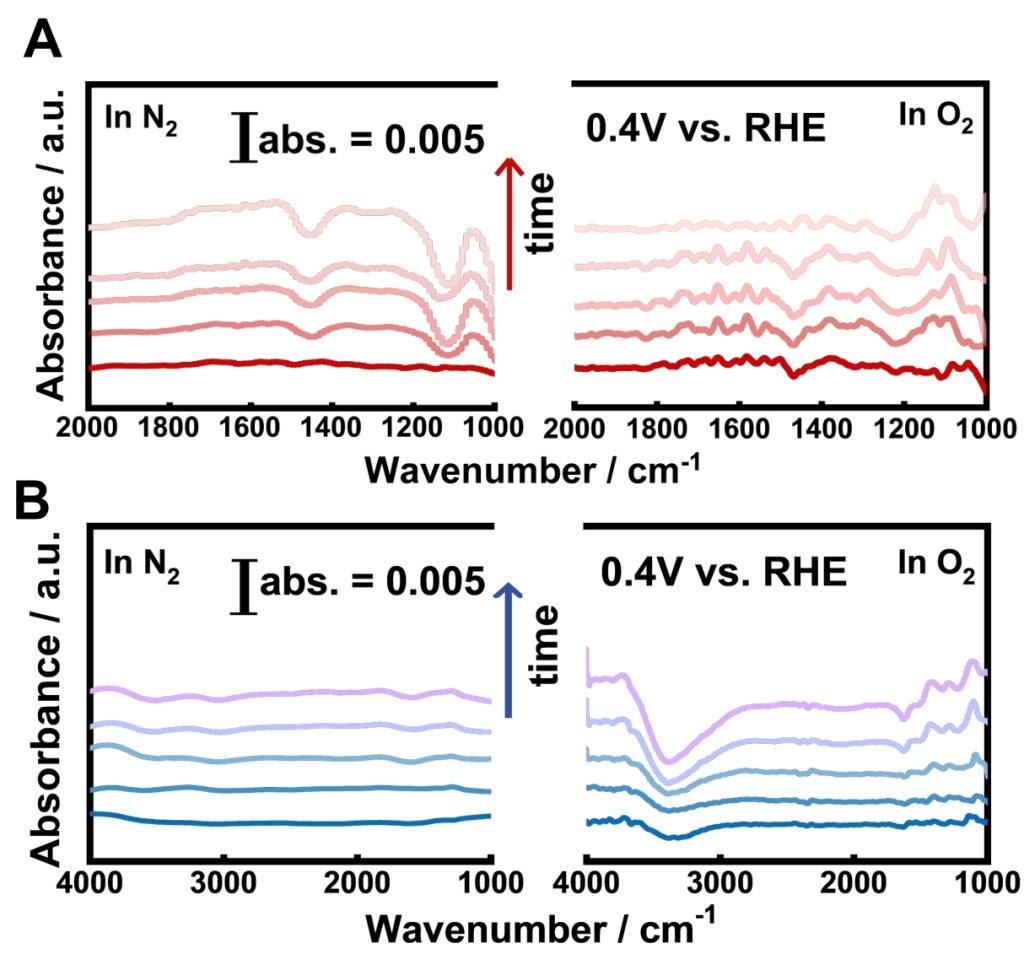


Figure S16. In situ FT-IR spectra of Ni/NG-1 (A) and Ni/NG-2 (B) during ORR in the presence and absence of O_2_.


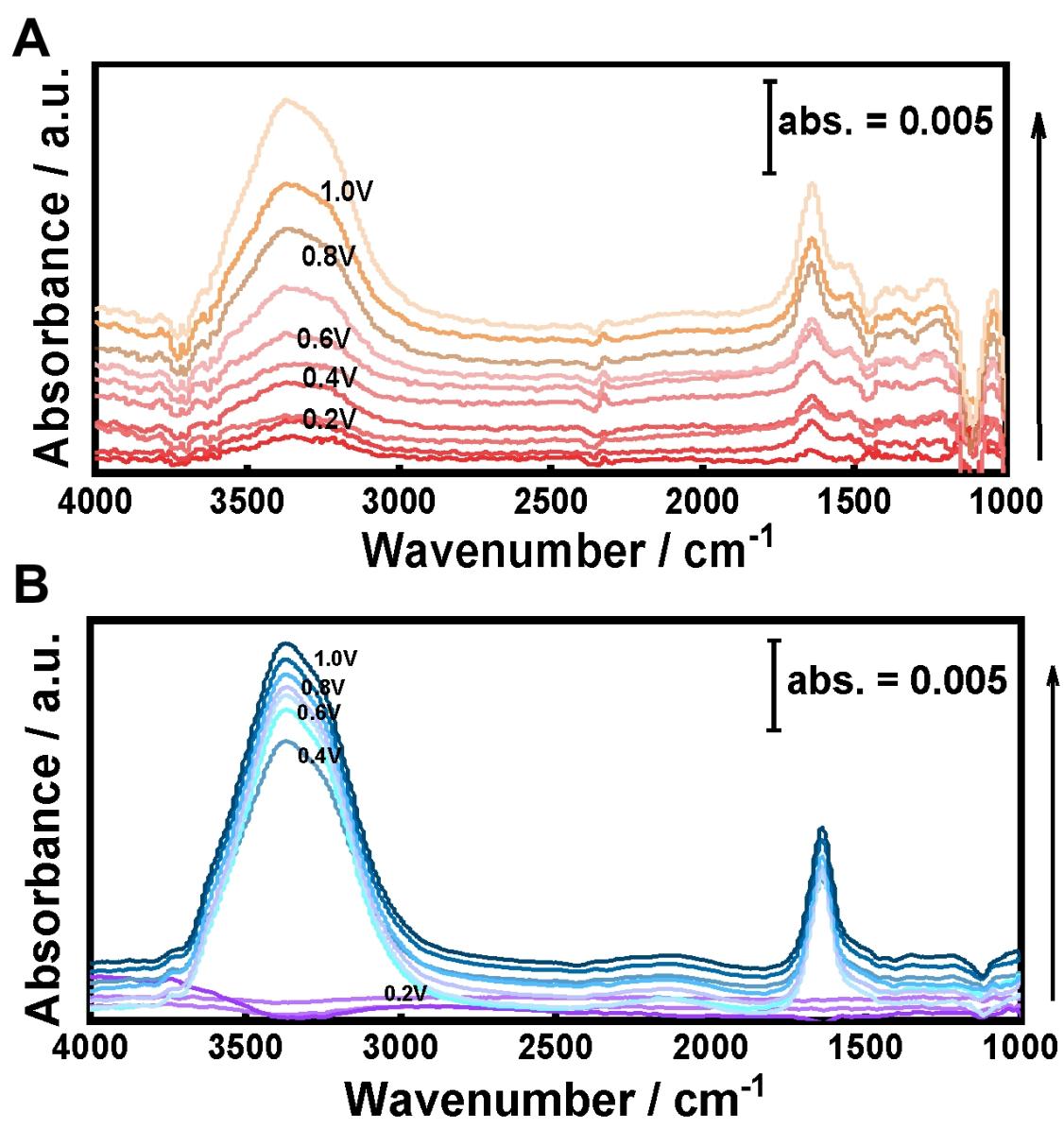


Figure S17. In situ FT-IR spectra of Ni/NG-1 (A) and Ni/NG-2 (B) during ORR at different reduction potential.


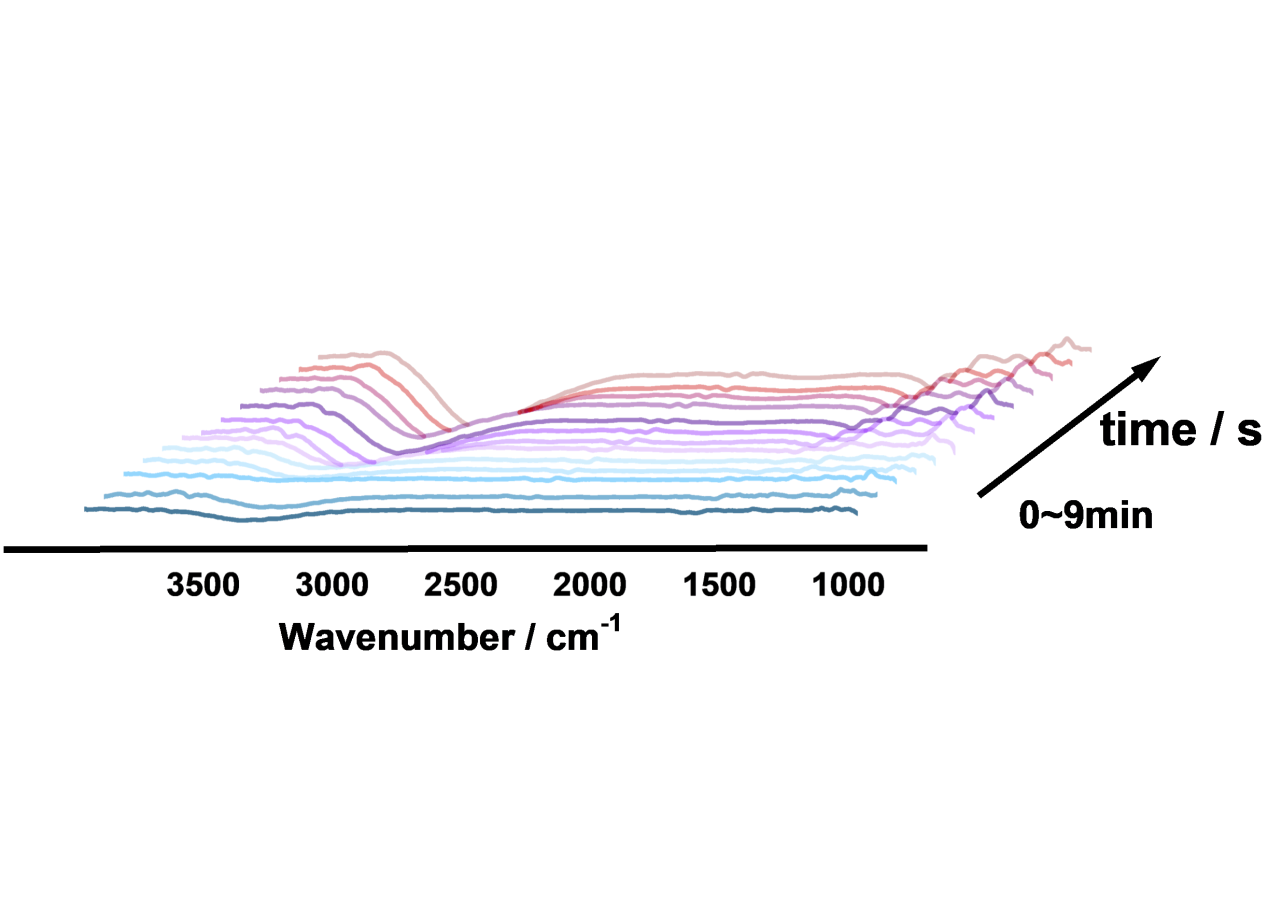


Figure S18. In situ FT-IR spectra of Ni/NG-2.


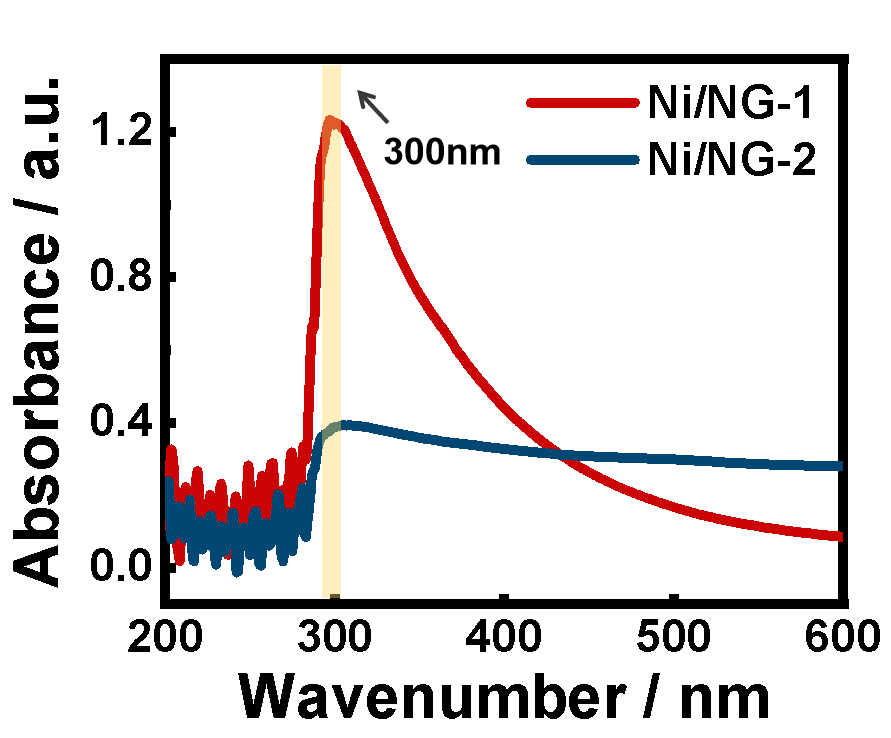


Figure S19. UV-vis of Ni/NG-1 and Ni/NG-2.


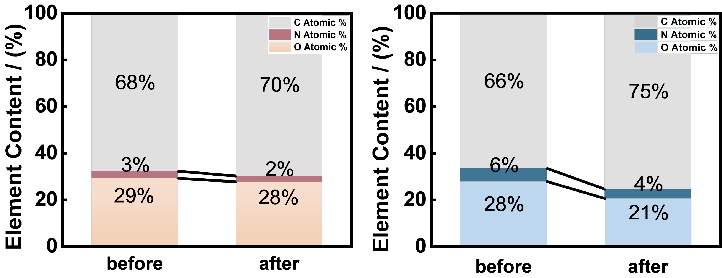


Figure S20. The C, N, and O elemental contents of Ni/NG-1 and Ni/NG-2 before and after ECL test determined by XPS.


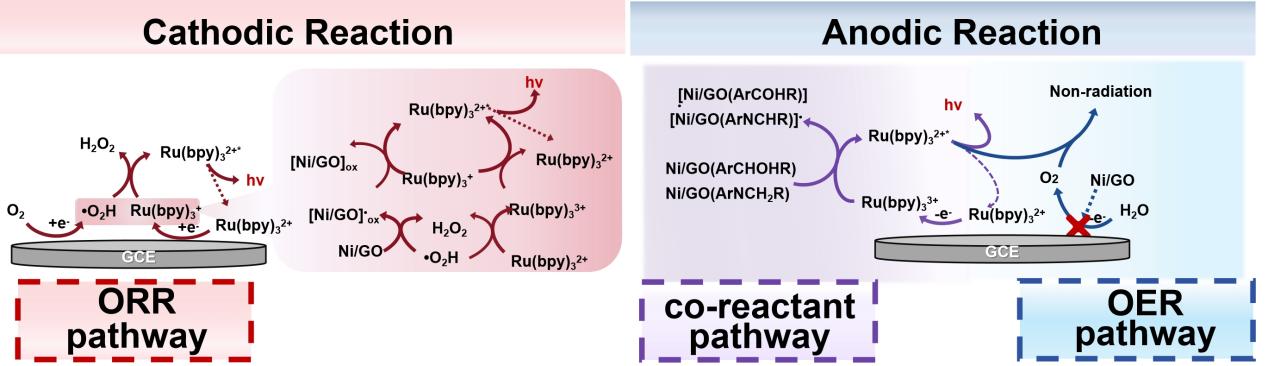


Figure S21. The luminous mechanism and transfer of electrons of Ni/NG catalysts.


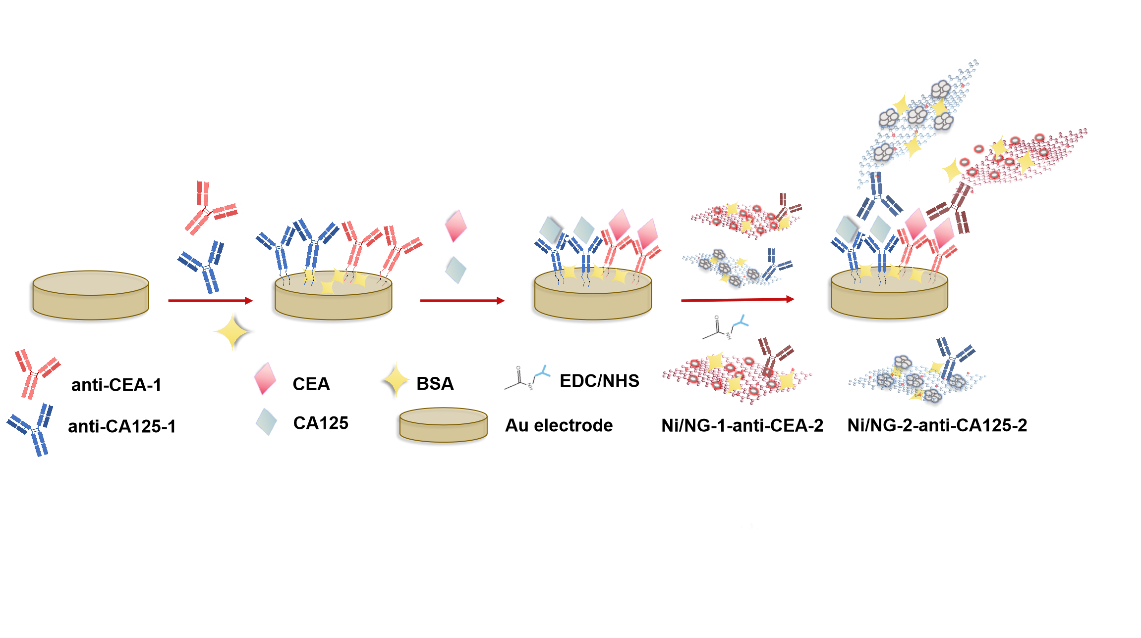


Figure S22. The construction of multi-marker ECL biosensor.


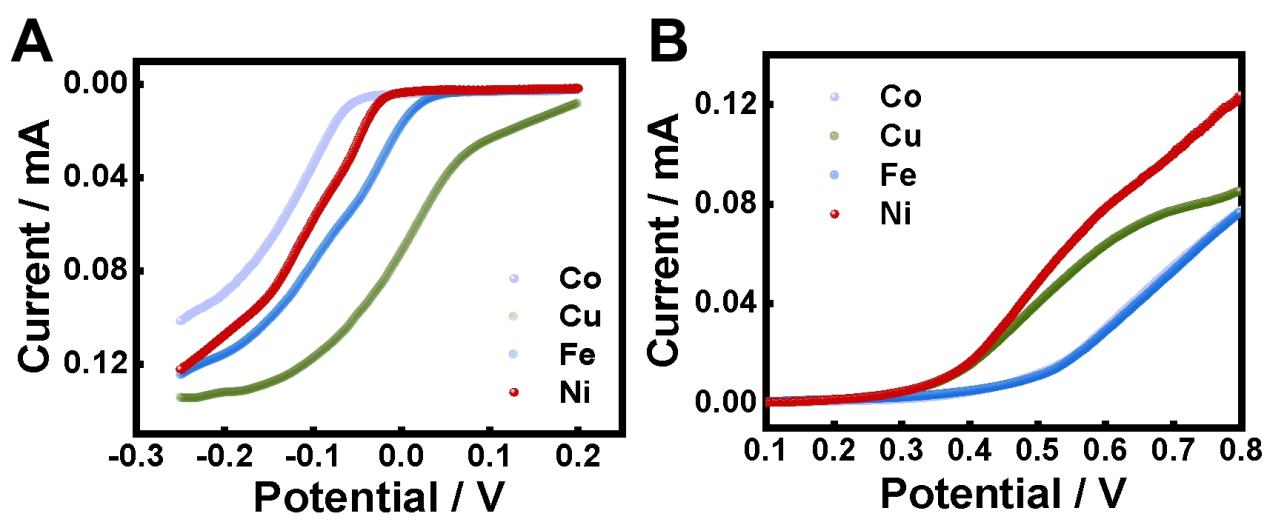


Figure S23. LSV curves of ORR (A) and OER (B) in 0.1 M KOH of Fe/NG, Ag/NG, and Cu/NG.


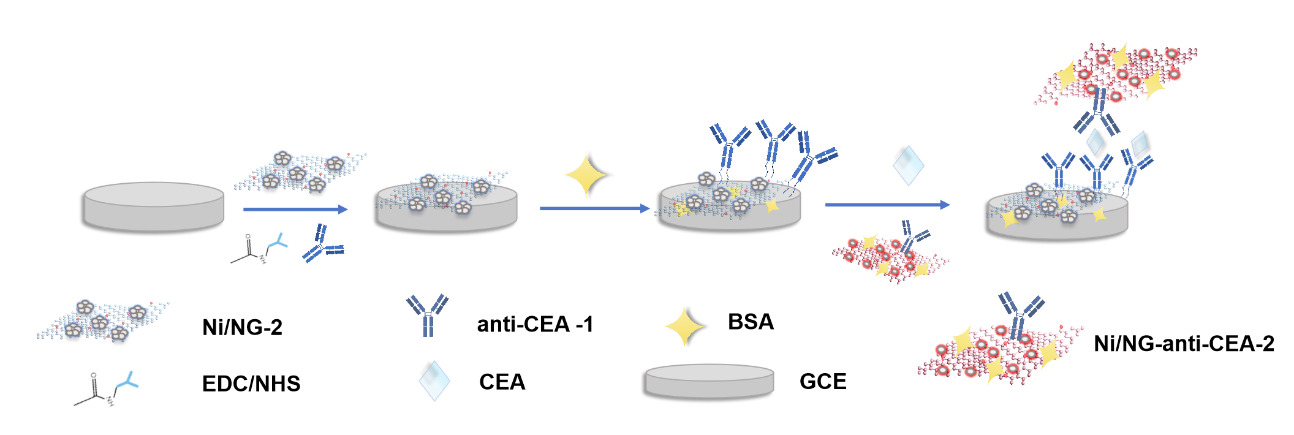


Figure S24. The construction of ratiometric immunosensor for single marker detection.


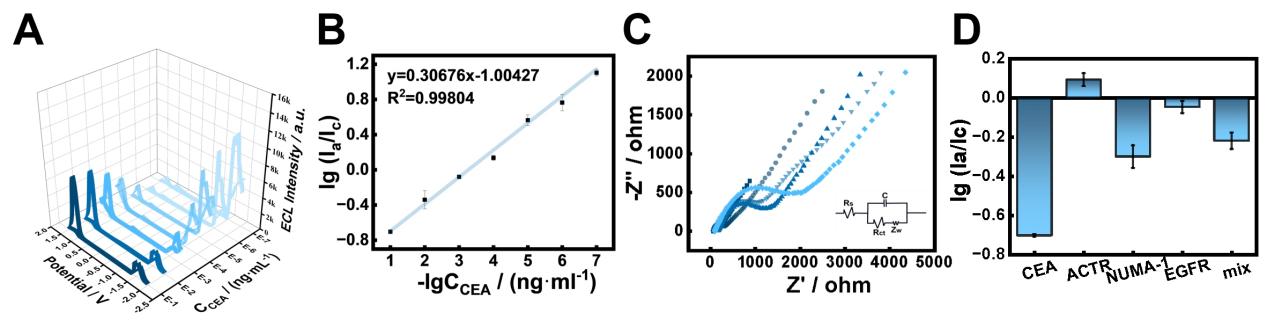


Figure S25. Detection performance of the proposed ratiometric ECL immunosensor. (A) ECL responses and (B) calibration plot of the ECL intensity with different concentrations of CEA from 10^-7^ ng mL^-1^ to 10^-1^ ng mL^-1^ in 1 mM Ru(bpy)_3_^2+^. (C) EIS. (D) The Selectivity.

| Element | Weight | Atomic % | Error % |
| --- | --- | --- | --- |
| C K | 44.74 | 48.71 | 4.75 |
| N K | 54.84 | 51.20 | 12.94 |
| Ni K | 0.42 | 0.09 | 60.59 |

| Element | Weight | Atomic % | Error % |
| --- | --- | --- | --- |
| C K | 41.86 | 53.06 | 7.66 |
| N K | 38.49 | 41.84 | 11.90 |
| Ni K | 19.65 | 5.10 | 5.33 |

Table S1. Mapping of Ni/NG-1 and Ni/NG-2.

It demonstrated that Ni/NG-1 shows 0.09% Ni content and Ni/NG-2 shows 5.10% Ni content.

| Element | C atomic % | N atomic % | O atomic % |
| --- | --- | --- | --- |
| before | 67.18 | 3.01 | 29.81 |
| after | 69.82 | 2.46 | 27.72 |

| Element | C atomic % | N atomic % | O atomic % |
| --- | --- | --- | --- |
| before | 29.81 | 3.01 | 67.18 |
| after | 27.72 | 2.46 | 69.82 |

Table S2. C, N, O content of Ni/NG-1 and Ni/NG-2 on the GCE before and after a 1-hour reaction with 1 mM Ru(bpy)_3_^2+^ (measured by XPS).

| Sample | Reference method | | Proposed method | | Relative error(%) | | Recovery(%) | |
| --- | --- | --- | --- | --- | --- | --- | --- | --- |
|  | CEA  (ng mL^-1^) | CA125  (U mL^-1^) | CEA  (ng mL^-1^) | CA125  (U mL^-1^) | CEA | CA125 | CEA | CA125 |
| 1 | 0.95 | 2560.00 | 1.04 | 2304.31 | 8.37 | -11.10 | 109.14 | 90.01 |
| 2 | 3.44 | 15.50 | 3.35 | 15.73 | -2.56 | 1.47 | 97.51 | 101.49 |
| 3 | 2.52 | 41.00 | 2.44 | 39.29 | -3.18 | -4.34 | 96.92 | 95.84 |
| 4 | 26.20 | 21.50 | 27.79 | 22.85 | 5.73 | 5.92 | 106.07 | 106.29 |
| 5 | 2.13 | 7.66 | 2.15 | 8.40 | 0.70 | 8.79 | 100.71 | 109.64 |
| 6 | 294.00 | 158.00 | 276.97 | 152.89 | -6.15 | -3.35 | 94.21 | 96.76 |

Table S3. Immunoassay of CEA and CA125 in clinical serum samples using our method^a^ and the reference method^b^.

a. Provided as the mean of 6 repeated tests. In our approach, the sera were diluted 10000-fold with PBS (pH = 7.4). RE, relative error.

b. The reference method was the commercialized ECLIA that uses the Elecsys CEA and Elecsys CA125, conducted on an electrochemiluminescence analyzer in the hospital.

| Sample | Reference method  (ng mL^-1^) | Propoesed method  (ng mL^-1^) | Relative error(%) | Recovery(%) |
| --- | --- | --- | --- | --- |
| 1 | 97.90 | 93.16 | -5.08 | 95.16 |
| 2 | 29.20 | 31.78 | 8.12 | 108.84 |
| 3 | 14.90 | 14.72 | -1.19 | 98.82 |
| 4 | 1.24 | 1.27 | 2.05 | 102.09 |
| 5 | 0.50 | 0.48 | -3.43 | 96.68 |
| 6 | 283.00 | 294.80 | 4.00 | 104.17 |

Table S4. Immunoassay of CEA in clinical serum samples using our method and the reference method.

a. Provided as the mean of 6 repeated tests. In our approach, the sera were diluted 10000-fold with PBS (pH = 7.4). RE, relative error.

b. The reference method was the commercialized ECLIA that uses the Elecsys CEA, conducted on an electrochemiluminescence analyzer in the hospital.

Equation S1

Surface of the electrode：

$${{Ru\left( bpy \right)}_{3}}^{2+} +e^{-}\to{{Ru\left( bpy \right)}_{3}}^{+}$$

$$O_{2} +e^{-}+H^{+}\to\bullet O_{2}H$$

$$\bullet O_{2}H+ e^{-} + H^{+}\to H_{2}O_{2}$$

Pathway 1：

•O_2_H / H_2_O_2_ oxidizes Ru(bpy)_3_^+^ to directly produce Ru(bpy)_3_^2+*^, thus promoting luminescence.

$${{Ru(bpy)}_{3}}^{+} + \bullet O_{2}H / H_{2}O_{2} + H^{+} \to{{Ru(bpy)}_{3}}^{2+*} + H_{2}O_{2} / \bullet OH \to{{Ru\left( bpy \right)}_{3}}^{2+} + hv$$

Pathway 2：

•O_2_H / H_2_O_2_ and Ni/NG generate Ni/NG radicals, and Ni/NG active radicals oxidize Ru(bpy)_3_^+^ and generate Ru(bpy)_3_^2+*^, subsequently promoting luminescence.

$$\bullet O_{2}H / H_{2}O_{2} + Ni/NG \to{[Ni/NG]}_{ox}\bullet+ H_{2}O_{2} / \bullet OH$$

$${{Ru(bpy)}_{3}}^{+}+ {[Ni/NG]}_{ox}\bullet+ H^{+} \to{{Ru(bpy)}_{3}}^{2+*} + Ni/NG \to{{Ru(bpy)}_{3}}^{2+}+ hv$$

Pathway 3：

·O_2_H / H_2_O_2_ oxidizes Ru(bpy)_3_^2+^ to Ru(bpy)_3_^3+^，and Ru(bpy)_3_^3+^ undergoes an annihilation reaction with Ru(bpy)_3_^+^ to produce the excited state Ru(bpy)_3_^2+*^ and promote luminescence.

$${{Ru(bpy)}_{3}}^{2+}+ \bullet O_{2}H / H_{2}O_{2} + H^{+} \to{{Ru(bpy)}_{3}}^{3+} + H_{2}O_{2} / \bullet OH$$

$${{Ru(bpy)}_{3}}^{3+} + {{Ru(bpy)}_{3}}^{+}\to{{Ru(bpy)}_{3}}^{2+*} + {{Ru(bpy)}_{3}}^{2+}\to{{Ru(bpy)}_{3}}^{2+} + hv$$

Equation S2

Ni/NG-2, its anodic ECL emission relies on the action of the OER product •OH and its own functional groups, both of which serve as co-reactants to ultimately generate Ru(bpy)_3_^2+*^. The equations for its luminescence are as follows:

Pathway 1：

The hydroxyl groups of Ni/NG-2 function as reaction sites. After single-electron oxidation, they transform into alkoxide radicals, which subsequently undergo deprotonation to form relevant reduction intermediates. These intermediates further reduce Ru(bpy)_3_^3+^ to Ru(bpy)_3_^2+*^, resulting in anodic ECL.

$${{Ru(bpy)}_{3}}^{2+} - e^{-} \to{{Ru(bpy)}_{3}}^{3+}$$

$$Ni/NG(ArCHOHR) -e^{-}\to{[Ni/NG(ArCHOHR)]}^{+\bullet} \to{[Ni/NG(ArCOHR)]}^{\bullet}$$

$${[Ni/NG(ArCOHR)]}^{\bullet} + {{Ru(bpy)}_{3}}^{3+} \to{{Ru(bpy)}_{3}}^{2+*} + Ni/NG(ArCOR) \to{{Ru(bpy)}_{3}}^{2+} + hv$$

Pathway 2：

The Amino groups of Ni/NG-2 serve as reaction sites. The reaction mechanism is similar to that of Ru(bpy)_3_^2+^-TPrA system, but it involves several distinct reaction pathways.10,11

Pathway 2-1：

When the applied potential is lower than the oxidation potential of Ru(bpy)_3_^2+^, Ni/NG(ArNRCH_2_R’) loses an electron on the electrode surface, generating [Ni/NG(ArNRCH_2_R’)]^+•^ with a certain oxidation capability. Subsequently, it loses a proton to form a Ni/NG(ArNRCHR’)^•^ radical, which reacts with Ru(bpy)_3_^2+^ to produce Ru(bpy)_3_^+^. Simultaneously, [Ni/NG(ArNRCH_2_R’)]^+•^ acts as an oxidant, converting Ru(bpy)_3_^+^ to Ru(bpy)_3_^2+*^, resulting in anodic ECL.

$$Ni/NG(ArNRCH_{2}R’) - e^{-}\to{[Ni/NG(Ar{NRCH}_{2}R’)]}^{+\bullet}$$

$${[Ni/NG(ArNRCH_{2}R’)]}^{+\bullet}- H^{+}\to{[Ni/NG(ArNRCHR’)]}^{\bullet}$$

$${[Ni/NG(ArNRCHR’)]}^{\bullet}+{{Ru(bpy)}_{3}}^{2+}\to{[Ni/NG(ArRN=CHR’)]}^{+}+ {{Ru(bpy)}_{3}}^{+}$$

$${[Ni/NG(ArNRCH2R’)]}^{+\bullet}+ {{Ru(bpy)}_{3}}^{+} \to{{Ru(bpy)}_{3}}^{2+*}+{[Ni/NG(ArNRCH_{2}R’)]}^{\bullet}\to{{Ru(bpy)}_{3}}^{2+}+hv$$

Pathway 2-2:

When the applied potential exceeds the oxidation potential of Ru(bpy)_3_^2+^, Ru(bpy)_3_^2+^ is directly electrochemically oxidized to Ru(bpy)_3_^3+^. The following mechanism can be explained by the timing of its interaction with Ni/NG(ArNCH_2_R):

At the electrode surface, Ru(bpy)_3_^2+^ undergoes electrooxidation to form Ru(bpy)_3_^3+^, while Ni/NG(ArNRCH_2_R’) is oxidized to [Ni/NG(ArNRCH_2_R’)]^+•^. After proton removal, [Ni/NG(ArNRCHR’)]^•^, a highly reducible species, is generated. [Ni/NG(ArNRCHR’)] ^•^ can then reduce Ru(bpy)_3_^3+^ to the excited state Ru(bpy)_3_^2+^.

$$Ni/NG(ArNRCH_{2}R’)-e^{-}\to{[Ni/NG(ArNRCH_{2}R’)]}^{+\bullet}$$

$${[Ni/NG(ArNRCH_{2}R’)]}^{+\bullet}-H^{+}\to{[Ni/NG(ArNRCHR’)]}^{\bullet}$$

$${[Ni/NG(ArNRCHR’)]}^{\bullet}+{{Ru(bpy)}_{3}}^{3+} \to{[Ni/NG(ArRN=CHR’)]}^{+}+{{Ru(bpy)}_{3}}^{2+*}\to{{Ru(bpy)}_{3}}^{2+}+hv$$

$${[Ni/NG(ArRN=CHR’)]}^{+}+H_{2}O- H^{+}\to Ni/NG(ArNRH) + R’CHO$$

Pathway 3：

The highly reducible [Ni/NG(ArNRCHR’)]^•^ in Pathway 2 can also reduce Ru(bpy)_3_^2+^ in the solution to form Ru(bpy)_3_^+^. Subsequently, Ru(bpy)_3_^+^ and Ru(bpy)_3_^3+^ undergo annihilation pathways to generate ECL.

$${[Ni/NG(ArNRCHR’)]}^{\bullet}+{{Ru(bpy)}_{3}}^{2+}\to{[Ni/NG(ArRN=CHR’)]}^{+}+{{Ru(bpy)}_{3}}^{+}$$

$${{Ru(bpy)}_{3}}^{+}+ {{Ru(bpy)}_{3}}^{3+}\to{{Ru(bpy)}_{3}}^{2+*}+{{Ru(bpy)}_{3}}^{2+}\to{{Ru(bpy)}_{3}}^{2+}+hv$$

Equation S3

OER reaction products promote anodic ECL.At the electrode surface, the generated •OH catalyzes the conversion of Ni/NG(ArNRCH_2_R’) / [Ni/NG(ArCOH_2_R)] into [Ni/NG(ArNRCH_2_R’)]^•^ / [Ni/NG(ArCOH_2_R)]^•^. These radicals then react with Ru(bpy)_3_^3+^ to produce excited state Ru(bpy)_3_^2+^.

Pathway 1:

$$H_{2}O-e^{-}\to H^{+}+ \bullet OH$$

$${{Ru(bpy)}_{3}}^{2+}-e^{-}\to{{Ru(bpy)}_{3}}^{3+}$$

$$[Ni/NG(ArNRCH_{2}R’)]/[Ni/NG(ArCOH_{2}R)]+ \bullet OH\to{[Ni/NG(ArNRCHR’)]}^{\bullet}/ {[Ni/NG(ArCOHR)]}^{\bullet}+H_{2}O$$

$${[Ni/NG(ArNRCHR’)]}^{\bullet}/{[Ni/NG(ArCOHR)]}^{\bullet}+{{Ru(bpy)}_{3}}^{3+}\to{{Ru(bpy)}_{3}}^{2+*}+{[Ni/NG(ArRN=CHR’)]}_{ox}/{[Ni/NG(ArC=OR)]}_{ox}+H^{+}\to{{Ru(bpy)}_{3}}^{2+}+ hv$$

$${{Ru(bpy)}_{3}}^{2+*}+O_{2}\to{{Ru(bpy)}_{3}}^{2+}+products [This side effect does not occur]$$

Pathway 2:

The •OH generated catalyzes the oxidation of -COOH, resulting in the formation of -COO• radicals and H_2_O. The -COO• radicals undergo decarboxylation to generate CO_2_ and Ni/NG• radicals. Ni/NG• then reduces Ru(bpy)_3_^3+^ to form excited-state Ru(bpy)_3_^2+*^.

$${{Ru\left( bpy \right)}_{3}}^{2+}-e^{-}\to{{Ru\left( b\mathrm{py} \right)}_{3}}^{3+}$$

$$Ni/NG\text{-}COOH+ \bullet OH\to{Ni/NG\text{-}COO}^{\bullet}+H_{2}O\to{Ni/NG}^{\bullet}+CO_{2}+ H_{2}O$$

$${Ni/NG}^{\bullet}+ {{Ru(bpy)}_{3}}^{3+}\to Ni/NG+ {{Ru(bpy)}_{3}}^{2+*}\to{{Ru(bpy)}_{3}}^{2+} + hv$$

Pathway 3:

The •OH catalyzes the oxidation of -COOH, leading to the formation of positively charged Ni/NG^+^ ions an**d negatively** charged CO_2_˙^−^ ions. The highly reducible CO_2_˙^−^ reduces Ru(bpy)_3_^3+^ to excited-state Ru(bpy)_3_^2+*^.

$${Ni/NG\text{-}COO}^{-}+ \bullet OH+H^{+}\to{Ni/NG}^{+}+ {{CO}_{2}}^{\cdot-}$$

$${{CO}_{2}}^{\cdot-}+{{Ru(bpy)}_{3}}^{3+}\to{{Ru(bpy)}_{3}}^{2+*}+CO_{2}\to{{Ru(bpy)}_{3}}^{2+} + hv$$

Reference

[1] a)X. Yang, J. Zhu, L. Qiu, D. Li, *Advanced Materials* 2011, 23, 2833; b)J. E. Kim, T. H. Han, S. H. Lee, J. Y. Kim, C. W. Ahn, J. M. Yun, S. O. Kim, *Angewandte Chemie International Edition* 2011, 50, 3043.

[2] H. Wang, T. Maiyalagan, X. Wang, *Acs Catalysis* 2012, 2, 781.

[3] a)V. H. Pham, T. V. Cuong, S. H. Hur, E. W. Shin, J. S. Kim, J. S. Chung, E. J. Kim, *Carbon* 2010, 48, 1945; b)S. Chen, J. Duan, J. Ran, M. Jaroniec, S. Z. Qiao, *Energy & Environmental Science* 2013, 6, 3693.

[4] a)A. Mohsenzadeh, K. Bolton, T. Richards, *Surface Science* 2014, 627, 1; b)H. Fang, S. Wu, T. Ayvali, J. Zheng, J. Fellowes, P.-L. Ho, K. C. Leung, A. Large, G. Held, R. Kato, K. Suenaga, Y. I. A. Reyes, H. V. Thang, H.-Y. T. Chen, S. C. E. Tsang, *Nature Communications* 2023, 14.
